# PBTK model-based analysis of CYP3A4 induction and the toxicokinetics of the pyrrolizidine alkaloid retrorsine in man

**DOI:** 10.1007/s00204-024-03698-2

**Published:** 2024-03-25

**Authors:** Anja Lehmann, Ina Geburek, Stefanie Hessel-Pras, Anne-Margarethe Enge, Hans Mielke, Christine Müller-Graf, Charlotte Kloft, Christoph Hethey

**Affiliations:** 1 German Federal Institute for Risk Assessment (BfR), Max-Dohrn-Str. 8-10, 10589 Berlin, Germany; 2 Department of Clinical Pharmacy and Biochemistry, Institute of Pharmacy, Freie Universitaet Berlin, 12169 Berlin, Germany

**Keywords:** PBPK model, Drug–drug interaction, Enzyme induction, Bioactivation, Rifampicin

## Abstract

Cytochrome P450 (CYP)3A4 induction by drugs and pesticides plays a critical role in the enhancement of pyrrolizidine alkaloid (PA) toxicity as it leads to increased formation of hepatotoxic dehydro-PA metabolites. Addressing the need for a quantitative analysis of this interaction, we developed a physiologically-based toxicokinetic (PBTK) model. Specifically, the model describes the impact of the well-characterized CYP3A4 inducer rifampicin on the kinetics of retrorsine, which is a prototypic PA and contaminant in herbal teas. Based on consumption data, the kinetics after daily intake of retrorsine were simulated with concomitant rifampicin treatment. Strongest impact on retrorsine kinetics (plasma AUC$_{24}$ and $C_{{\text {max}}}$ reduced to 67% and 74% compared to the rifampicin-free reference) was predicted directly after withdrawal of rifampicin. At this time point, the competitive inhibitory effect of rifampicin stopped, while CYP3A4 induction was still near its maximum. Due to the impacted metabolism kinetics, the cumulative formation of intestinal retrorsine CYP3A4 metabolites increased to 254% (from 10 to 25 nmol), while the cumulative formation of hepatic CYP3A4 metabolites was not affected (57 nmol). Return to baseline PA toxicokinetics was predicted 14 days after stop of a 14-day rifampicin treatment. In conclusion, the PBTK model showed to be a promising tool to assess the dynamic interplay of enzyme induction and toxification pathways.

**Supplementary Information:**

The online version contains supplementary material available at 10.1007/s00204-024-03698-2.

## Introduction

Retrorsine is a potent hepatotoxic pyrrolizidine alkaloid (PA) found in plants of the *Senecio* genus in various regions of the world (Dimande et al. 2007; Johnson et al. 1989). Humans are exposed to PAs by the intake of PA-containing herbal medicines and food supplements or by the ingestion of PA-contaminated food, such as spices, tea and honey. Based on food consumption data from 2005 to 2006, 22% of German adults were identified as consumers of herbal tea (Dusemund et al. 2018; MRI 2008). Analyses of European and German tea samples showed that retrorsine is one of the most frequently detected PAs in herbal tea along with senecionine and seneciphylline (Mulder et al. 2018; Schulz et al. 2015).

Cytochrome P450 (CYP)-mediated metabolic activation is required for most PAs to exert their hepatoxic, genotoxic and carcinogenic effects (Mattocks 1968). The active hepatic uptake of PAs is mediated via organic cation transporter 1 (OCT1) and sodium/taurocholate co-transporting polypeptide (NTCP) (Enge et al. 2021; Tu et al. 2014; Enge et al. 2022). Intracellular dehydrogenation via CYP enzymes forms reactive toxic metabolites, termed dehydro-PAs. Dehydro-PAs may bind to cellular proteins and nucleic acids yielding protein and DNA adducts, which are considered as biomarkers for PA exposure (Ma et al. 2018; He et al. 2017). Based on in vitro studies with human CYP supersomes, the isoforms CYP3A4, CYP3A5 and CYP2A6 have been identified primarily involved in PA bioactivation (Ruan et al. 2014).

Inherent to their toxification mechanism, PAs are the ‘victim’ of CYP-mediated interactions, while compounds that alter the kinetics of the victim compound via CYP induction or inhibition are termed ‘perpetrators’, according to definitions in EMA (2012). Especially CYP inducers could play a central role as perpetrators in the enhancement of PA toxicity as they lead to an increased formation of toxic PA metabolites. Already in 1972, it was shown that rats pretreated with the CYP3A4-inducing drug phenobarbital prior to administration of the PA monocrotaline developed more severe toxic effects compared to absence of the pretreatment (Allen et al. 1972). In this study, a maximum 1.5-fold increase of relative amounts of dehydro-PA metabolites (3.7−5.7 optical density per unit liver wet weight) was observed in the liver of rats 1.5 h after subcutaneous injection of monocrotaline as a consequence of the induction of microsomal enzymes.

Besides phenobarbital, more than 20 widely used drugs are known perpetrators to induce CYP3A4 such as the antibiotic rifampicin, the antidepressant St. John’s wort, the antiretroviral HIV drug efavirenz, as well as anti-inflammatory glucocorticoids (Flockhart et al. 2021). However, a quantitative analysis of the CYP-mediated toxicokinetic interaction between drugs and PA is not established in humans.

In addition to drugs, a number of pesticides including fipronil, chlorpyrifos, permethrin and dichlorodiphenyltrichloroethane (DDT) have been shown to be inducers of CYP isoforms in vitro (Hodgson and Rose 2007). HepG2 cells treated with DDT showed an increase of CYP3A4 gene expression by factor 13 (Medina-Díaz and Elizondo 2005). Treatment of human hepatocytes with fipronil resulted in up to 30-fold induction of CYP3A4 mRNA levels (Das et al. 2006).

In view of the increased PA toxicity via CYP-mediated interactions, there is a need to pay attention to combination effects in risk assessment of PAs. Physiologically-based toxicokinetic (PBTK) or pharmacokinetic (PBPK) modeling is a powerful tool to explore and quantitatively predict kinetic interactions (Hanke et al. 2018). The physiology-based approach allows to species-specifically integrate enzyme and transporter levels of interest into the liver and the intestine compartment of a PBTK model.

Due to lacking mechanistic and quantitative knowledge of PA–drug interactions in humans, the aim of this study was to predict the kinetics and the magnitude of the interaction between the PA representative retrorsine and the CYP3A4 inducer rifampicin. The antibiotic rifampicin is a potent inducer of multiple drug metabolizing enzymes and transporters. The drug is recommended for induction studies of CYP3A4 (US Food and Drug Administration 2023), since its strong induction effect on CYP3A4 is well-characterized and it is not a substrate to the enzyme itself (Flockhart et al. 2021). Effects of rifampicin on hepatic protein levels of the uptake transporters OCT1 and NTCP have not been reported. Therefore, no transporter-mediated interaction between rifampicin and retrorsine is expected.

The PBTK model for retrorsine alone previously developed and evaluated in rodents was extrapolated to the human anatomy, physiology and biochemistry (Lehmann et al. 2023). Retrorsine exposure was simulated based on PA consumption data for herbal tea and was combined with the intake of a therapeutic dose of rifampicin. Based on the mode of action of retrorsine bioactivation via hepatic and intestinal CYP3A4 enzymes, formation of retrorsine CYP3A4 metabolites was selected as internal metric to assess the effect of rifampicin.

## Methods

### PBTK model of retrorsine

**Fig. 1:**
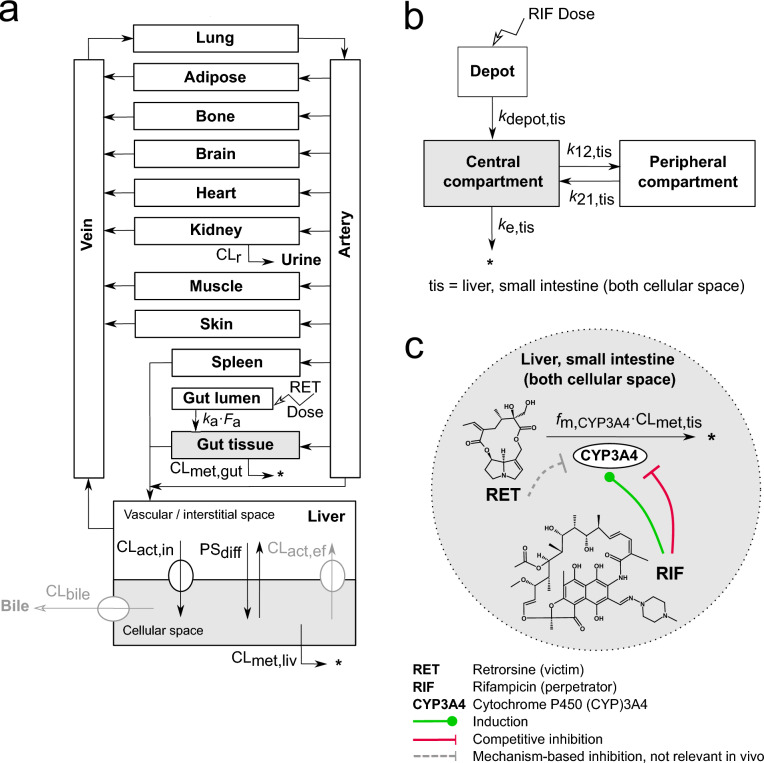
**a** Physiologically-based toxicokinetic (PBTK) model of retrorsine. The liver compartment was represented by the extended clearance model accounting for hepatic transport and metabolism. **b** Empirical 2-compartment model of rifampicin parametrized for liver (intracellular) and small intestine (intracellular), separately. **c** Retrorsine-rifampicin interaction mediated by CYP3A4 induction and competitive CYP3A4 inhibition by rifampicin. All parameters of the retrorsine-rifampicin interaction model are summarized in Tables S1–S2. The full system of ordinary differential equations is given in Eqs. S1–S15. CL$_{\text {act,in}}$ Active uptake clearance; CL$_{\text {act,ef}}$ Active efflux clearance; CL$_{\text {bile}}$ Biliary clearance; CL$_{\text {met,gut}}$ Gut metabolic clearance; CL$_{\text {met,liv}}$ Liver metabolic clearance; CL$_{\text {met,tis}}$ Liver or gut metabolic clearance; CL$_{\text {r}}$ Renal clearance; $F_{\text {a}}$ Intestinal fraction absorbed; $f_{{\textrm{m}},{\textrm{CYP}}3A4}$ Fraction of retrorsine metabolized by CYP3A4; *k*_12,tis_ Transition rate constant from central to peripheral compartment for rifampicin; *k*_21,tis_ Transition rate constant from peripheral to central compartment for rifampicin; $k_{\text {a}}$ Intestinal absorption rate constant; $k_{\text {depot,tis}}$ Absorption rate constant from depot compartment for rifampicin; $k_{\text {e,tis}}$ Elimination rate constant for rifampicin; PS$_{\text {diff}}$ Passive influx diffusion flow rate; tis = liver cellular space or small intestine (both cellular space)

The PBTK model structure (Fig. 1a) was adopted from our previously published mouse and rat PBTK model of retrorsine (Lehmann et al. 2023). Based on the substrate affinity of retrorsine toward the previously described hepatic uptake transporters, the extended clearance model of the liver was integrated considering hepatic transport in addition to hepatic metabolism (Patilea-Vrana and Unadkat 2016; Schweinoch 2014; Sirianni and Pang 1997).

Anatomy- and physiology-related model parameters, such as tissue volumes V$_{\text {tis}}$ and blood flows Q$_{\text {tis}}$, were updated from rodents to the standard male human of 73 kg body weight with average age of 35 years (ICRP 2002). Tissue distribution of retrorsine was described via predicted tissue-to-plasma partition coefficients K$_{\text {tis}}$ (Rodgers and Rowland 2006). Collection of physiological parameters as well as prediction of K$_{\text {tis}}$ was based on a pharmacometric modeling framework provided by Hartung and Huisinga (2019).

Biochemical parameters characterizing hepatic uptake and metabolism of retrorsine were determined experimentally and scaled via in vitro-to-in vivo extrapolation. Passive diffusion PS$_{\text {diff}}$ and transporter-mediated uptake CL$_{\text {act,in}}$ into the liver cell were quantified from medium loss assays with cultivated HepaRG cells at non-physiological (4 $^\circ$C) and physiological (37 $^\circ$C) temperature, respectively. Measurements from medium loss assays were previously published by Enge et al. (2021). We described the time-dependent depletion of retrorsine in the incubation medium with a monoexponential model (Eq. S16). The rate constants $\lambda _{4^\circ \text {C}}$ and $\lambda _{37^\circ \text {C}}$, used for calculation of PS$_{\text {diff}}$ and CL$_{\text {act,in}}$ (Eqs. S17–S20), were estimated using Markov Chain Monte Carlo (MCMC) analysis. Of note, CL$_{\text {act,in}}$ describes the phenomenological uptake by all transporters of that retrorsine is a substrate including OCT1 and NTCP. Individual kinetics of distinct uptake transporters as well as saturation of transport were not included in the PBTK model. For a detailed protocol of the medium loss assay, see Supplementary Information (‘Culture of HepaRG cells’ and ‘Medium loss assay’).

Liver metabolic clearance CL$_{\text {met,liv}}$ was determined using human liver microsomes with different retrorsine concentrations to account for saturation of metabolism (for detailed protocol see Supplementary Information ‘Liver microsomal assay’). We described the time- and concentration-dependent depletion of retrorsine in liver microsomes by the end-product inhibition model (Eqs. S21–S24), which assumes irreversible inhibition of CYP enzymes by reactive dehydro-PA metabolites in vitro. The maximum reaction velocity $V_{\text {max,liv,in vitro}}$, the Michaelis–Menten constant $K_{\text {M,liv}}$ and the concentration at half-maximal inhibition $\text {IC}_{50}$ of the end-product inhibition model were estimated using MCMC. This end-product inhibition of retrorsine is relevant in closed in vitro systems including liver microsomes as shown for CYP3A4 (Dai et al. 2010). However, in vivo it was assumed to be not relevant, because dynamic flows constantly remove reactive metabolites. This assumption was shown to be valid for mouse hepatic kinetics of retrorsine (Lehmann et al. 2023).

Active efflux clearance CL$_{\text {act,ef}}$ and biliary clearance CL$_{\text {bile}}$ were assumed negligible based on observations in rodents (see Lehmann et al. (2023)). Overall liver clearance CL$_{\text {liv}}$ of retrorsine was calculated according to the extended clearance model (Patilea-Vrana and Unadkat 2016):

1 $$\begin{aligned} \text {CL}_{\text {liv}}= \, \frac{ Q_{\text {liv}} \cdot f_{\text {u,p}} \cdot \text {CL}_{\text {act,in}} \cdot (\text {CL}_{\text {met,liv}} + \text {CL}_{\text {bile}})}{Q_{\text {liv}} \cdot (\text {CL}_{\text {act,ef}} + \text {CL}_{\text {met,liv}} + \text {CL}_{\text {bile}}) + f_{\text {u,p}} \cdot \text {CL}_{\text {act,in}} \cdot (\text {CL}_{\text {met,liv}} + \text {CL}_{\text {bile}}) } \end{aligned}$$

with Q$_{\text {liv}}$ = hepatic blood flow, f$_{\text {u,p}}$ = fraction unbound in plasma, CL$_{\text {act,in}}$ = active influx clearance, CL$_{\text {met,liv}}$ = liver metabolic clearance (ratio of $V_{\text {max,liv}}$ to $K_{\text {M,liv}}$), $V_{\text {max,liv}}$ = maximum metabolism reaction velocity, $K_{\text {M,liv}}$ = concentration at half-maximal metabolism reaction velocity, CL$_{\text {bile}}$ = biliary clearance, CL$_{\text {act,ef}}$ = active efflux clearance.

Included extrahepatic elimination routes were intestinal metabolism and renal excretion. Glomerular filtration was considered the predominant mechanism of renal clearance CL$_{\text {r}}$ as suggested in rodents (Lehmann et al. 2023). Regarding intestinal metabolism, $K_{\text {M,gut}}$ and $V_{\text {max,gut}}$ were specified in accordance with a comparative analysis of hepatic and intestinal metabolic activity of CYP3A substrates in vitro (Galetin and Houston 2006). The intestinal Michaelis–Menten constant was assumed to be identical to that of hepatic metabolism ($K_{\text {M,gut}}=K_{\text {M,liv}}$). The maximum reaction velocity $V_{\text {max,gut}}$ was assumed to be smaller by factor 10 compared to $V_{\text {max,liv}}$ of hepatic metabolism and confirmed by sensitivity analysis indicating similar model predictions. In addition, we introduced a volume fraction of gut tissue $F_{\text {si}}$ that represents the cellular space of the small intestine to confine retrorsine gut metabolism to the site of CYP3A4 expression (Eq. S8).

Oral uptake of retrorsine was modeled as first-order absorption process from the gut lumen to the gut tissue. The intestinal absorption rate constant $k_{\text {a}}$ was estimated previously based on kinetic data from an oral dose study in mice (Lehmann et al. 2023). The fraction absorbed from the gut $F_{\text {a}}$ was calculated previously making use of a correlation between Caco-2 cell permeability and human intestinal absorption, which was reported for a set of 93 diverse compounds (Lehmann et al., 2023; Skolnik et al., 2010).

The full system of ordinary differential equations of the PBTK model of retrorsine is given in Eqs. S1–S12 with all model parameters shown in Table S1.

### 2-Compartment model of rifampicin

For prediction of rifampicin kinetics a previously published PBPK model was used (Hanke et al. 2018). The model was available in the Open Systems Pharmacology (OSP) Suite (https://www.open-systems-pharmacology.org/, accessed 3 April 2023) and was evaluated for the prediction of CYP3A4-mediated interactions. Rifampicin uptake transport by organic anion transporting polypeptide 1B1 (OATP1B1), metabolism by acrylacetamide deacetylase (AADAC), efflux transport by P-glycoprotein (P-gp) and glomerular filtration were included in the model. Furthermore, auto-induction of OATP1B1, AADAC and P-gp expression were considered.

The OSP Suite and the R framework (see Methods section ‘Software’) have a different compartmentalization of liver and gut. To obtain comparable rifampicin concentration-time profiles, rifampicin kinetics from the OSP Suite needed a scaled integration in the R framework of this study. More specific, the OSP Suite differentiated between vascular, interstitial and cellular space for both tissues. The presented R framework has for the liver a lumped vascular/interstitial separated from a cellular space. The gut tissue represents the vascular, interstitial and cellular space of both the large and the small intestine, all together. As the cellular space of the small intestine is the site of intestinal CYP3A4 expression, the intracellular small intestine concentrations of the OSP model were considered as the most relevant concentrations and therefore integrated in the R framework as gut tissue concentrations. The observed rifampicin concentrations of the cellular space of liver and small intestine were scaled with the corresponding tissue volumes (Table S1, liver cellular space, gut tissue) to account for minor volume differences between the OSP Suite/R software (i.e. liver cellular space 1.59 L vs. 1.30 L, gut tissue 1.13 L vs. 1.02 L). A 2-compartment disposition model with first-order absorption was fitted to the scaled concentration-time profiles of liver and small intestine (both intracellular), respectively (Fig. 1b and Eqs. S13–S15). The rate constants $k_{\text {depot,tis}}$, *k*_12,tis_, *k*_k21,tis_ and $k_{\text {e,tis}}$ were estimated via MCMC. Model evaluation plots and estimated parameters are provided as Supplementary Information (‘Evaluation of the 2-compartment model of rifampicin’, Fig. S1 and Table S2).

### Modeling of the CYP3A4-mediated interaction

To predict the CYP3A4-mediated interaction, induction and simultaneous competitive inhibition of the enzyme by rifampicin, were integrated in the model (Fig. 1c, green and red arrows). CYP3A4 induction was modeled by addition of an induction process in the CYP3A4 turnover equation, exemplarily shown for the liver cellular space (subscript liv, superscript c):

2 $$\begin{aligned} \begin{aligned} \frac{d\text {CYP3A4}_{\text {liv}}^{\text {c}}(t)}{{\textrm{d}}t}&= \underbrace{ R_{\text {syn}} }_\text {Synthesis rate} \cdot \Big (1+ \underbrace{ \frac{E_{{\text {max}}} \cdot \frac{\text {RIF}_{\text {liv}}^{\text {c}}(t)}{V_{\text {liv}}^{\text {c}}}}{\text {EC}_{50}+ \frac{\text {RIF}_{\text {liv}}^{\text {c}}(t)}{V_{\text {liv}}^{\text {c}}}} }_\text {Induction} \Big ) - \underbrace{ k_{\text {deg}} \cdot \text {CYP3A4}_{\text {liv}}^{\text {c}}(t) }_\text {Degradation rate} \\ R_{\text {syn}}&= k_{\text {deg}} \cdot \text {CYP3A4}_{0,\text {liv}}^{\text {c}} \end{aligned} \end{aligned}$$

with $d\text {CYP3A4}_{\text {liv}}^{\text {c}}(t)/dt$ = change of CYP3A4 amount in liver cellular space over time *t*, $R_{\text {syn}}$ = rate of CYP3A4 synthesis in absence of inducer (steady-state assumption), $k_{\text {deg}}$ = degradation rate constant, $\text {CYP3A4}_{0,\text {liv}}^{\text {c}}$ = CYP3A4 baseline amount, $V_{\text {liv}}^{\text {c}}$ = volume of the liver cellular space, $E_{{\text {max}}}$ = maximal induction effect of rifampicin in vivo, $\text {EC}_{50}$ = rifampicin concentration at half-maximal induction in vivo, $\text {RIF}_{\text {liv}}^{\text {c}}(t)$ = rifampicin amount.

The induction process was characterized by $\text {EC}_{50} = 0.340\,\upmu$mol/L (corrected for protein binding) and $E_{{\text {max}}} = 9.00$ based on CYP3A4 induction in human primary hepatocytes (Hanke et al. 2018; Templeton et al. 2011; Shou et al. 2008).

Competitive inhibition of CYP3A4 by rifampicin was modeled by addition of an inhibitor process on the Michaelis–Menten constant $K_{\text {M}}$ of the cellular retrorsine metabolism as follows:

3 $$\begin{aligned} \begin{aligned} \frac{d\text {RET}_{\text {liv}}^{\text {c}} (t)}{{\textrm{d}}t}&= \ldots - f_{\text {m,CYP3A4}} \cdot \frac{k_{\text {cat,CYP3A4,liv}} \cdot \text {CYP3A4}_{\text {liv}}^{\text {c}}(t)}{K_{\text {M,liv}} \cdot \Big (1+ \underbrace{ \frac{\text {RIF}_{\text {liv}}^{\text {c}}(t)}{V_{\text {liv}}^{\text {c}}} \cdot \frac{1}{K_{i}} }_{\begin{array}{c} {\text {Competitive}} \\ {\text {inhibition}} \end{array}} \Big ) + f_{\text {u,liv}}^{\text {c}} \cdot \frac{\text {RET}_{\text {liv}}^{\text {c}}(t)}{V_{\text {liv}}^{\text {c}}}} \cdot f_{\text {u,liv}}^{\text {c}} \cdot \frac{\text {RET}_{\text {liv}}^{\text {c}}(t)}{V_{\text {liv}}^{\text {c}}} \end{aligned} \end{aligned}$$

with $d\text {RET}_{\text {liv}}^{\text {c}} (t)/{\textrm{d}}t$ = change of retrorsine amount in the liver cellular space over time *t*, $f_{\text {m,CYP3A4}}$ = fraction of retrorsine metabolized by CYP3A4, $k_{\text {cat,CYP3A4,liv}}$ = catalytic constant, $\text {CYP3A4}_{\text {liv}}^{\text {c}}(t)$ = CYP3A4 amount, $K_{\text {M,liv}}$ = retrorsine concentration at half-maximal reaction velocity, $\text {RIF}_{\text {liv}}^{\text {c}}(t)$ = rifampicin amount, $V_{\text {liv}}^{\text {c}}$ = volume of liver cellular space, $K_{i}$ = rifampicin concentration at half-maximal inhibition, $f_{\text {u,liv}}^{\text {c}}$ = fraction unbound in liver cellular space.

The competitive inhibition process was parametrized by $K_{\text {i}} = 18.5\,\upmu$mol/L, as determined in human liver microsomes (Hanke et al. 2018; Kajosaari et al. 2005).

Equivalently to the liver, the interaction was implemented in the gut (Eqs. S8 and S12). The retrorsine PBTK model and the hepatic and intestinal rifampicin two-compartment model were linked and their ordinary differential equations were solved simultaneously. Mechanism-based inhibition of CYP3A4 by reactive dehydro-PA metabolites in vitro (Dai et al. 2010) (Fig. 1c, grey dashed arrow) was not integrated in the PBTK model since it was assumed to be not relevant in vivo (see Methods section ‘PBTK model of retrorsine’).

### Fraction of retrorsine metabolized by CYP3A4

The fraction of retrorsine metabolized by CYP3A4 $f_{\text {m,CYP3A4}}$ was derived from in vitro assays with individual human recombinant CYP supersomes (Ruan et al. 2014). Graphical data reporting the concentration of protein adducts formed from oxidation of retrorsine by individual CYP isoforms were extracted using the online tool WebPlotDigitizer (Rohatgi 2020). The following protein adduct concentrations (nM) were obtained: 34.2 (CYP1A1), 47.4 (CYP1A2), 451 (CYP2A6), 387 (CYP3A4), 292 (CYP3A5), 132 (CYP2B6), 71.1 (CYP2C9), 123 (CYP2C19), 153 (CYP2D6), 63.6 (CYP2E1). Protein adduct concentrations were scaled linearly with the abundance of hepatic CYP isoforms in vivo (pmol/mg): 10 (CYP1A1), 39 (CYP1A2), 27 (CYP2A6), 93 (CYP3A4), 17 (CYP3A5), 16 (CYP2B6), 61 (CYP2C9), 11 (CYP2C19), 12.6 (CYP2D6), 64.5 (CYP2E1) (Achour et al. 2014; Klomp et al. 2020). The resulting percentage of retrorsine-derived protein adducts formed by CYP3A4 among the sum of all scaled protein adducts was 52.7%. Under the assumption that the fraction of retrorsine metabolized by CYP3A4 is equal to the fraction of retrorsine-derived protein adducts formed by CYP3A4, $f_{\text {m,CYP3A4}}$ was set to 0.527. Integration of $f_{\text {m,CYP3A4}}$ into the PBTK model allowed the simulation of CYP3A4 metabolites of retrorsine. Accordingly, the fraction of non-CYP3A4 metabolites of retrorsine formed was modeled as 1-$f_{\text {m,CYP3A4}}$.

### Simulated exposure scenarios

In the scenario ‘herbal tea’, the daily consumption of herbal tea with a retrorsine dose of 0.019 $\upmu$g/kg body weight/day was simulated for 5 weeks. In the scenario, ‘herbal tea + rifampicin’, the daily consumption of this herbal tea was combined with a 2-week daily therapeutic dose (start at day 4) of 600 mg rifampicin orally. Population median consumption of herbal tea was taken from Dusemund et al. (2018) (Table 5, ‘Herbal tea not specified’, Median intake). 600 mg of rifampicin per day correspond to the recommended therapeutic dose for the treatment of tuberculosis (van Ingen et al. 2011).

The retrorsine dose in both exposure scenarios was set equal to the total PA dose of published consumption data, since no PBTK models are available for all different types of PAs and PA *N*-oxides that can be found in herbal tea.

### Sensitivity analysis

A sensitivity analysis was performed to identify the influence of the interaction model parameters $f_{\text {m,CYP3A4}}$, $\text {EC}_{50}$, $E_{{\text {max}}}$ and $K_{\text {i}}$ on the formation of hepatic and intestinal retrorsine CYP3A4 metabolites at five weeks of simulation. To test sensitivity, the four parameters were reduced by 10% and increased by 10%. The corresponding sensitivity coefficients were calculated as follows:

4 $$\begin{aligned} {\text {Sensitivity coefficient}} = \frac{\Delta A/A_{0}}{\Delta p/p_{0}} \end{aligned}$$

with $\Delta A$ = the difference between the resulting and original ($A_{0}$) predicted cumulative concentration of CYP3A4 metabolites at 5 weeks of simulation, $\Delta p$ = the difference between the resulting and initial ($p_{0}$) parameter values.

### Software

All modeling activities were performed within the software R Version 4.3.2 (R Core Team 2023) and RStudio Version 2023.09.1.494 (Posit Team 2023). Parameter estimates of MCMC (R packages dfoptim, Varadhan et al. (2020) and FME, Soetaert and Petzoldt (2010)) were reported as mode and (95% credible interval) of the posterior distribution (R package bayestestR, Makowski et al. (2019)). Ordinary differential equations were solved using the R package rxode2 (Fidler et al. (2023)). Figures were generated by the R package ggplot2 (Wickham 2016) and finalized within InkScape (InkscapeProject 2020).

## Results

### Predicted human retrorsine kinetics

#### Oral absorption, protein binding and tissue distribution

A high fraction of retrorsine ($F_{\text {a}} = 78.3\%$) is moderately fast absorbed by the gut tissue ($k_{\text {a}} = 0.910$ 1/h) as determined previously in vitro (Lehmann et al. 2023). The fraction unbound in plasma is high (fuP = 60%) as was determined previously via rapid equilibrium dialysis (Haas et al. 2019). Tissue-to-plasma partition coefficients $K_{\text {tis}}$ predicted for humans by the Rodgers and Rowland model revealed that retrorsine is most weakly distributed into adipose ($K_{\text {adi}} = 0.222$) and bone ($K_{\text {bon}} = 0.625$) and most strongly distributed into the lung tissue ($K_{\text {lun}} = 1.11$). All predicted $K_{\text {tis}}$ are listed in Table S1.

#### Hepatic elimination based on the extended clearance model

In the extended clearance model of the liver, hepatic elimination is described as an interplay of transport and metabolism.

**Fig. 2:**
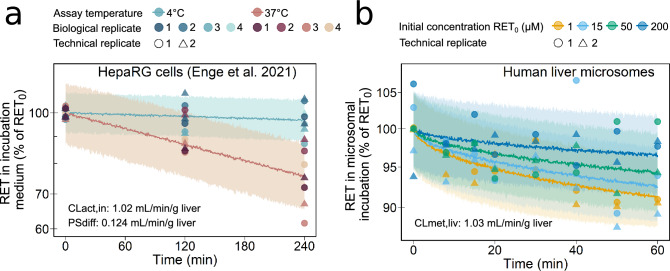
**a** Temperature-dependent retrorsine (RET) depletion in incubations of HepaRG cells determined by Enge et al. (2021). Medium loss assays were performed either at 4 $^\circ$C or at 37 $^\circ$C. Observed data ($n=4$ biological replicates, each assessed with $n=2$ technical replicates) were described with a monoexponential model (Eq. S16). Median (solid lines) and 5–95% interpercentile range (shaded areas) are based on 1000 Monte Carlo simulations. In vivo liver active uptake CL$_{\text {act,in}}$ and passive diffusion PS$_{\text {diff}}$ were predicted for non-saturating conditions (Eqs. S17–S20). **b** Concentration-dependent RET depletion in human liver microsomal incubations. Microsomal assays were performed with 1, 15, 50 or 200 $\upmu$M of retrorsine (RET$_0$). Observed data ($n=2$ technical replicates using microsomes of pooled livers) were described by an end-product inhibition model (Eqs. S21–S24). Median (solid lines) and 5–95% interpercentile range (shaded areas) are based on 1000 Monte Carlo simulations. In vivo liver metabolic clearance CL$_{\text {met,liv}} = V_{\text {max,liv}}/K_{\text {M,liv}}$ was predicted for the case of linear kinetics (RET $\ll K_{\text {M,liv}}$)

The active uptake clearance $\text {CL}_{\text {act,in}}$ (mL/min/g liver) of 1.02 predicted from $\lambda _{37^\circ \text {C}}$ in HepaRG cells showed that transporter-mediated uptake is the main route of membrane permeation (Fig. 2a, Table 1 and Eqs. S17–S20). The passive influx diffusion flow rate $\text {PS}_{\text {diff}}$ (mL/min/g liver) of 0.124 predicted from $\lambda _{4^\circ \text {C}}$ in HepaRG cells was eight times smaller.

Retrorsine taken up into the liver cellular space is metabolized with a predicted metabolic clearance CL$_{\text {met,liv}}$ (mL/min/g liver) of 1.03 derived from the ratio of the maximum reaction velocity $V_{\text {max,liv}}$ of 26.2 nmol/min/g liver to the Michaelis–Menten constant $K_{\text {M,liv}}$ of 25.5 $\upmu$M, both predicted from assays with human liver microsomes (Fig. 2b and Table 1). The concentration- and time-dependent depletion of retrorsine in microsomal incubations was adequately described by the end-product inhibition model (Fig. 2b and Eqs. S21–S24). Low dose exposure of herbal tea intake are within the linear range of the end-product inhibition model. In the end-product inhibition model irreversible inhibition of biotransformation enzymes by reactive retrorsine metabolites was assumed as was shown in vitro with CYP3A4 (Dai et al. 2010). The product concentration IC$_{50}$, at which retrorsine biotransformation was inhibited half-maximal, was predicted to be small with a value of $5.18\cdot 10^{-4}$ $\upmu$M (Table 1). End-product inhibition was modeled in vitro, but assumed absent in vivo (see Methods section ‘PBTK model of retrorsine’).

Hepatic efflux of retrorsine was assumed negligible. According to the extended clearance model (Eq. 1), overall liver clearance CL$_{\text {liv}}$ was 9.08 mL/min/kg body weight.

**Table 1:** Parameter estimates of the monoexponential model (medium loss assay, Eq. S16) and the end-product inhibition model (microsomal assay, Eqs. S21–S24)

Parameter	Unit	Posterior mode (95% credible interval)	
*Monoexponential model*			
$\lambda _{4^\circ \text {C}}$	1/h	0.00754	[0.00115, 0.0156]
$\lambda _{37^\circ \text {C}}$	1/h	0.0696	[0.0574, 0.0840]
*End-product inhibition model*			
IC$_{50}$	$\upmu$M	$5.18\cdot 10^{-4}$	[$1.24\cdot 10^{-4}$, $5.78\cdot 10^{-3}$]
$V_{\text{max,liv, in vitro}}$	$\upmu$M/min	0.817	[0.219, 9.03]
$V_{\text {max,liv}}{^{\dagger }}$	nmol/min/g liver	26.2	[7.01, 289]
$K_{\text {M,liv}}$	$\upmu$M	25.5	[7.55, 98.6]

$^{\dagger }$Predicted by in vitro-to-in vivo extrapolation (Eq. S25)

#### Extrahepatic elimination

Extrahepatic elimination routes included in the PBTK model of retrorsine were renal excretion and intestinal metabolism. Renal clearance CL$_{\text {r}}$ via glomerular filtration was predicted as 0.9 mL/min/kg body weight. Intestinal metabolic clearance CL$_{\text {met,gut}}$ (ratio of $V_{\text {max,gut}}$ and $K_{\text {M,gut}}$) was determined as 1.35 mL/min/kg body weight.

#### Biodistribution and bioavailability

Biodistribution for retrorsine doses up to 100 $\upmu$g/kg body weight was predicted as follows. After rapid absorption from the gut lumen, the highest retrorsine fractions were predicted in muscle (14%) and bone (2%), while the lowest retrorsine fractions were predicted in spleen, kidneys and heart (< 0.2%) (Fig. S2). In plasma, retrorsine peaked to 0.9% of the administered dose. 99.99% of retrorsine were eliminated from the body after 17 h. At this time point, liver metabolism accounted for 79% of retrorsine elimination, while 13% of retrorsine were metabolized by the gut tissue. 8% of the dose were excreted renally.

Oral bioavailability *F* of retrorsine was predicted 44%. *F* was mostly affected by hepatic metabolism (fraction escaping hepatic first-pass metabolism $F_{\text {h}} = 60\%$), followed by intestinal absorption (fraction absorbed from the gut tissue $F_{\text {a}} = 78\%$) and less affected by gut metabolism (fraction escaping gut first-pass metabolism $F_{\text {g}} = 94\%$).

### Predicted CYP3A4-mediated interaction

#### CYP3A4 enzyme kinetics

At the start of the simulation of the daily oral administration of 600 mg rifampicin, the relative concentration of hepatic and intestinal CYP3A4 rapidly increased (Fig. S3). A maximal fold increase of 10 in the liver and of 9 in the intestine was predicted at day 1 after the last dose of rifampicin. This increase took 17 days after the last dose of rifampicin for CYP3A4 levels to return to baseline.

#### Retrorsine kinetics

For herbal tea in absence of rifampicin, area-under-the-curve in 24-h intervals (AUC$_{24}$) of retrorsine were 0.0732, 0.0468 and 0.140 nmol/L$\cdot$h and maximum tissue concentrations $C_{{\text {max}}}$ were 0.0178, 0.0148 and 0.0538 nmol/L in plasma, liver and gut tissue, respectively (Table 2, ‘Herbal tea’ exposure scenario). Retrorsine concentrations in liver and gut tissue were rate-limiting to the interaction with rifampicin, since $C_{{\text {max}}}$ values were very low compared to the Michaelis–Menten constant $K_{\text {M}}$ of 25.5 $\upmu$M.

**Fig. 3:**
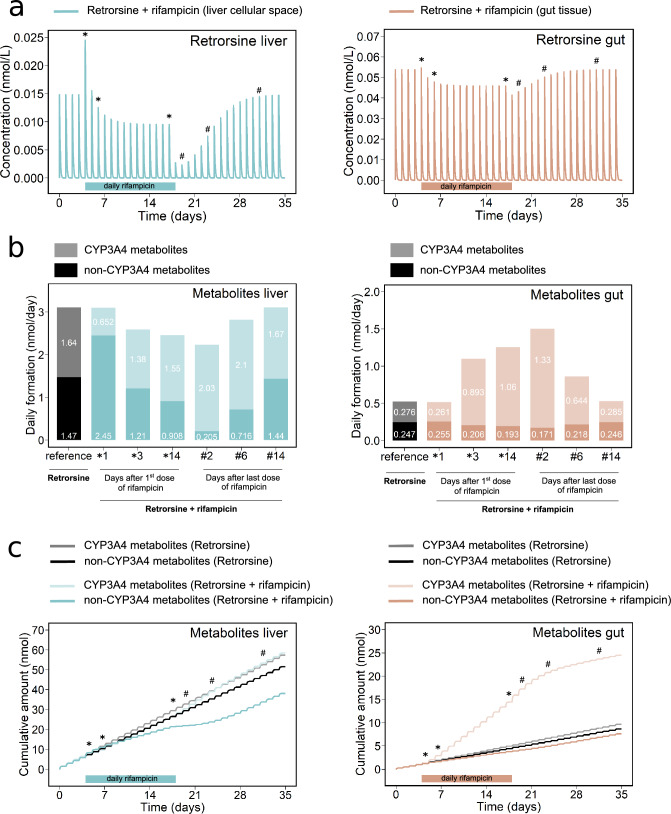
Predicted effect of a two-week oral intake of rifampicin (600 mg/day) on the kinetics of retrorsine during consumption of herbal tea with a retrorsine dose of 0.019 $\upmu$g/kg body weight/day for 5 weeks. **a** Concentration-time profiles of retrorsine in liver and gut tissue. **b** Daily formation of hepatic and intestinal CYP3A4 and non-CYP3A4 metabolites of retrorsine. **c** Time-dependent formation of hepatic and intestinal CYP3A4 and non-CYP3A4 metabolites of retrorsine given as cumulative amount. Note: Day 1, 3 and 14 after the first dose of rifampicin (CYP3A4 induction) are marked by $*$. Day 2, 6 and 14 after the last dose of rifampicin (CYP3A4 de-induction) are marked by #

At day 1 after the first dose of rifampicin, AUC$_{24}$ and $C_{{\text {max}}}$ values of retrorsine increased as a consequence of the instantaneous, but weak competitive inhibitory effect of rifampicin on CYP3A4 (Fig. 3a). Until day 14 after the first dose of rifampicin, AUC$_{24}$ and $C_{{\text {max}}}$ notably decreased due to the built-up of the counteracting induction effect of rifampicin on CYP3A4.

Strongest reduction of AUC$_{24}$ and $C_{{\text {max}}}$ was predicted after removal of rifampicin, when the competitive inhibitory effect stopped while CYP3A4 induction was still near its maximum. At day 2 after the last dose of rifampicin, AUC$_{24}$ of retrorsine was reduced to 67%, 14% and 69% and $C_{{\text {max}}}$ was reduced to 74%, 16% and 80% of the rifampicin-free reference in plasma, liver cellular space and gut tissue, respectively. According to the model, retrorsine kinetics normalized after 14 days of rifampicin discontinuation.

**Table 2:** Predicted area-under-the-curve in 24-h intervals (AUC$_{24}$) and maximum concentration ($C_{{\text {max}}}$) of retrorsine concentration-time profiles in plasma, liver cellular space and gut tissue

Exposure scenario	Predicted AUC$_{24}$ (nmol/L h)			Predicted $C_{{\textrm{max}}}$ (nmol/L)		
	Plasma	Liver	Gut	Plasma	Liver	Gut
Herbal tea	0.0732	0.0468	0.140	0.0178	0.0148	0.0538
*Herbal tea + rifampicin*$^{\dagger }$						
Days after first dose of rifampicin						
$*$1	0.0771	0.0780	0.144	0.0188	0.0245	0.0547
$*$3	0.0609	0.0386	0.116	0.0158	0.0125	0.0478
$*$14	0.0567	0.0289	0.109	0.0149	0.00952	0.0458
Days after last dose of rifampicin						
#2	0.0491	0.00650	0.0965	0.0131	0.00235	0.0430
#6	0.0637	0.0228	0.124	0.0160	0.00746	0.0502
#14	0.0729	0.0458	0.139	0.0178	0.0145	0.0537

The daily consumption of herbal tea with a retrorsine dose of 0.019 $\upmu$g/kg body weight/day was simulated in absence and in presence of the daily intake of 600 mg rifampicin

$^{\dagger }$600 mg of rifampicin, p.o. daily for 14 days; Day 1, 3 and 14 after the first dose of rifampicin (CYP3A4 induction) are marked by $*$; Day 2, 6 and 14 after the last dose of rifampicin (CYP3A4 de-induction) are marked by #

#### Kinetics of CYP3A4 and non-CYP3A4 metabolites of retrorsine

For herbal tea in absence of rifampicin, the daily formation of CYP3A4 and non-CYP3A4 metabolites was constant and 6-fold higher in the liver compared to gut (Fig. 3b, grey and black bars). The cumulative amount of both metabolites linearly increased over the simulation time span of 5 weeks (Fig. 3c, grey and black lines).

At day 1 after the first dose of rifampicin, daily production of CYP3A4 metabolites decreased due to the instantaneous competitive inhibitory effect of rifampicin on CYP3A4 (Fig. 3b, colored bars).

Until day 14 after the first dose of rifampicin, daily formation of hepatic CYP3A4 metabolites increased but was still reduced compared to the rifampicin-free reference. Daily formation of intestinal CYP3A4 metabolites markedly increased during the built-up of the CYP3A4 induction effect of rifampicin.

The most pronounced increase in CYP3A4 metabolite formation was predicted after the last dose of rifampicin, when the competitive inhibitory effect stopped, while CYP3A4 induction was still strong. Daily formation of CYP3A4 metabolites increased 4.8-fold in the gut and 1.3-fold in the liver at day 2 and 6 after the last dose of rifampicin, respectively.

The daily production of CYP3A4 and non-CYP3A4 metabolites normalized after 14 days of rifampicin discontinuation.

Rifampicin intake resulted in increase of the cumulative amount of intestinal CYP3A4 metabolites to 254% (from 9.66 to 24.5 nmol) at 5 weeks (Fig. 3c, light orange vs. grey line). The cumulative amount of hepatic CYP3A4 metabolites almost remained constant (changed from 57.4 to 58.4 nmol) of the rifampicin-free reference (Fig. 3c, light blue vs. grey line).

### Sensitivity analysis

**Fig. 4:**
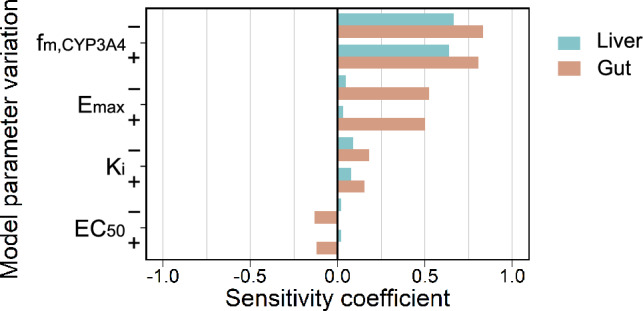
Sensitivity analysis of interaction model parameters. The sensitivity coefficient indicates the effect of parameter reduction by 10% (${-}$) and parameter increase by 10% (+) on the cumulative amount of retrorsine CYP3A4 metabolites in liver (blue color) and gut (orange color) at 5 weeks of model simulation. Abbreviations: $f_{\text {m,CYP3A4}}$ = fraction of retrorsine metabolized by CYP3A4, EC$_{50}$ = rifampicin concentration at half-maximal CYP3A4 induction in vivo, $E_{{\text {max}}}$ = maximal CYP3A4 induction effect of rifampicin in vivo, $K_{i}$ = rifampicin concentration at half-maximal CYP3A4 inhibition (color figure online)

The sensitivity coefficients of interaction model parameters are displayed in Fig. 4. Hepatic and intestinal retrorsine CYP3A4 metabolites were most sensitive to the fraction of retrorsine metabolized by CYP3A4 $f_{\text {m,CYP3A4}}$. Additionally, intestinal retrorsine CYP3A4 metabolites were sensitive to the maximal CYP3A4 induction effect of rifampicin $E_{{\text {max}}}$. Variation of the rifampicin concentration at half-maximal CYP3A4 inhibition $K_{i}$ or at half-maximal CYP3A4 induction EC$_{50}$ did not have any relevant effect on model predictions.

## Discussion

In this study, we predicted the kinetics and the magnitude of the CYP3A4-mediated interaction between the PA retrorsine and the CYP3A4 inducer rifampicin. Based on PA consumption data for herbal tea, daily retrorsine exposure was simulated in man and combined with a two-week intake of the antibiotic rifampicin. Simulations were performed within a PBTK model-based framework including the formation of retrorsine CYP3A4 and non-CYP3A4 metabolites.

Predicted tissue concentration-time profiles of retrorsine followed a distinct pattern over the course of rifampicin intake and after its removal. This kinetic pattern resulting from the overlapping processes of competitive CYP3A4 inhibition and induction is generalizable for all victim compounds of the CYP3A4-mediated interaction with rifampicin.

In vivo interaction studies with rifampicin as well as biomonitoring data are not available for retrorsine and PAs in general. However, the drug midazolam is a well-characterized victim of the CYP3A4-mediated interaction with rifampicin (Nassar et al. 2022). The pattern of de-induction kinetics predicted for retrorsine is comparable to that observed in vivo for midazolam. Reduction of retrorsine plasma AUC$_{24}$ and $C_{{\text {max}}}$ (reduced to 57% and 64% of reference) was not as pronounced as for midazolam (AUC$_{\infty }$ and $C_{{\text {max}}}$ reduced to 13% and 16% of reference) (Reitman et al. 2011). This is plausible since only 53% of retrorsine were assumed to be metabolized by CYP3A4, while midazolam is almost exclusively metabolized by the enzyme (Patki et al. 2003; Ruan et al. 2014).

Maximum hepatic and intestinal CYP3A4 enzyme activity was predicted to increase 10- and 9-fold, respectively. This prediction is comparable to that of previously published rifampicin PBPK models with equivalent dosing regimens (8- and 7-fold in liver and duodenum, Hanke et al. (2018) and 5.5- and 6.5-fold in liver and jejunum, Baneyx et al. (2014)). CYP3A4 levels were predicted to return to baseline within a period of two weeks after rifampicin discontinuation as in Baneyx et al. (2014) and Hanke et al. (2018).

Based on the mode of action of retrorsine bioactivation, formation of retrorsine CYP3A4 metabolites was selected as internal metric for the toxicokinetic interaction. To this end, we performed simulations for the CYP3A4 induced state with 0.019 $\upmu$g/kg body weight/day administered for 35 days (in total 138 nmol) of retrorsine. The results showed that the cumulative amount of CYP3A4 metabolites increased 2.5-fold from 10 to 25 nmol in the gut and remained constant at 57 nmol in the liver as a result of rifampicin intake compared to the rifampicin-free reference. Within the PBTK model, these differences are attributed to the gut first-pass effect, in which the liver is exposed only to the fraction of retrorsine that escapes gut metabolism. In the induced state, this fraction becomes smaller. At the same time, the induction shifts the equilibrium between daily CYP3A4 and non-CYP3A4 metabolite formation. Since the increase in the daily CYP3A4 metabolites formation was not enough to outweigh the changes in the first-pass effect in the inducted state, the cumulative amount of hepatic CYP3A4 metabolites remained constant in the induced state. Regarding the effective level of induction, note that the maximum rifampicin concentrations were approximately 7-fold higher in liver (140 $\upmu$mol/L) compared to gut (20 $\upmu$mol/L) (Fig. S1). Thus, for both tissues the induction threshold (EC$_{50} = 0.34\,\upmu$mol/L) was constantly exceeded. This full coverage explained the 10- and 9-fold relative induction of CYP3A4 seen in liver and gut as described above. Despite the high CYP3A4 induction, retrorsine concentrations in liver and gut tissue were rate-limiting to the interaction ($C_{{\text {max}}} \ll K_{\text {M}}$).

Importantly, CYP3A4 catalyzes PA bioactivation to toxic reactive metabolites, but also PA detoxification via *N*-oxidation and glutathione conjugation. A previous study, elucidating the metabolic pattern of retrorsine in CYP3A4-overexpressing HepG2 cells, found that of all identifiable metabolites retrorsine *N*-oxide was most abundant in the cell culture medium (Geburek et al. 2021). However, an unknown fraction, which was slightly higher compared to the fraction of identifiable metabolites, was observed for retrorsine and also for other PAs. It was suggested that the unknown fraction may comprise reactive metabolites that were retained within the cells covalently bound to biomolecules.

It needs to be highlighted that the fraction of retrorsine metabolized by CYP3A4 $f_{\text {m,CYP3A4}}$ used in the PBTK model is based on reported measurements of protein adduct formation in individual CYP supersomes that we scaled with in vivo CYP abundances (see section ‘Fraction of retrorsine metabolized by CYP3A4’) (Ruan et al. 2014). Sensitivity analysis has confirmed that model predictions of CYP3A4 metabolites are highly sensitive toward variation of $f_{\text {m,CYP3A4}}$. To increase accuracy of $f_{\text {m,CYP3A4}}$, retrorsine depletion instead of protein adduct formation could be measured in assays with individual CYP supersomes.

Liver microsomal assays showed that the metabolic clearance of retrorsine in humans was 2-fold higher compared to mice and 2-fold lower compared to rats (Lehmann et al. 2023). Medium loss assays revealed a 2-fold higher and a similar hepatic active uptake clearance of retrorsine in HepaRG cells compared to primary mouse and rat hepatocytes, respectively (Lehmann et al. 2023). Differentiated HepaRG cells were used as in vitro model since they stably express functional sinusoidal and canalicular transporters and CYP amounts comparable to human primary hepatocytes (Kanebratt and Andersson 2008; Tascher et al. 2019). The liver was predicted to act as a sink, since the sum of retrorsine elimination via metabolism and biliary excretion was notably higher compared to sinusoidal efflux. Both active uptake and metabolism were determined to be rate-limiting in the hepatic elimination of retrorsine.

A limitation of this study is the assumption that the retrorsine dose in the simulated exposure scenarios is equal to the total PA dose of published consumption data. Usually, combinations of several PAs and PA *N*-oxides can be found in teas (Mulder et al. 2015). However, since PBTK models are only available for few PAs, modeling of combined PA kinetics remains a challenge. In addition, a better understanding of potential kinetic interactions of individual PAs is required.

Retrorsine was identified as mechanism-based inhibitor of CYP3A4 in vitro with a half-maximal inhibitory concentration $K_{\text {I}}$ of 7.7 $\upmu$M and an inactivation rate constant $k_{\text {inact}}$ of 0.025 min$^{-1}$ (Dai et al. 2010). The mechanism-based inhibition of CYP enzymes by reactive dehydro-PA metabolites is relevant in closed systems such as liver microsomes. However, in vivo it was assumed to be not relevant due to the constant removal of reactive metabolites by dynamic flows, and was, therefore, not included in the PBTK model. This assumption was verified for mouse hepatic retrorsine kinetics previously (Lehmann et al. 2023). The in vitro observed CYP3A4 inhibitory capacity of retrorsine nevertheless would not be relevant for the simulated exposure scenarios, since maximum hepatic retrorsine concentrations of herbal tea consumption were five orders of magnitude below the reported $K_{\text {I}}$ value.

The role of retrorsine in the induction of CYP3A4 gene expression, which is mediated by activation of the pregnane X receptor (PXR), has not yet been conclusively determined. Reporter gene analysis with the human embryonic kidney cell line HEK-293 did not show any activation of PXR by retrorsine (Luckert et al. 2018). In contrast, Dai et al. (2021) observed activation of PXR by retrorsine in reporter gene assays with the human hepatic cell lines HepG2 and LO2. However, an increase of CYP3A4 gene expression was only determined in LO2 cells, but not in HepG2 cells. Further confirmatory assessment and a quantitative analysis of retrorsine’s role in CYP3A4 induction need to be performed to ensure a reliable integration into the PBTK model.

In conclusion, the PBTK-based approach was successfully applied to simulate the kinetics and magnitude of the CYP3A4-mediated interaction between the PA retrorsine and the antibiotic rifampicin in man. On this example, it was demonstrated that the PBTK model is a promising tool to evaluate the impact of CYP3A4 induction on toxification pathways. During antibiotic treatment as well as in clinical studies with rifampicin as CYP3A4 inducer, herbal tea consumption should be critically assessed during intake of rifampicin until 2 weeks after the last dose. Perspectively, it also needs to be assessed to which extent other CYP3A4 inducers such as pesticides, diet and drugs other than rifampicin affect toxicokinetics of retrorsine and other PAs.

## Supplementary Information

Below is the link to the electronic supplementary material.

Supplementary file 1 (pdf 744 KB)

## Data Availability

All data, material and code are available in the GitHub repository https://github.com/al901010/Supplement_PBTK_Interaction_Retrorsine_Rifampicin.
