# Supplementary material for: PBTK model-based analysis of CYP3A4 induction and the toxicokinetics of the pyrrolizidine alkaloid retrorsine in man
Source: Arch Toxicol. 2024 Mar 25;98(6):1757–69. doi: 10.1007/s00204-024-03698-2 (PMC11636752; doi:10.1007/s00204-024-03698-2)
Supplement: Supplementary file 1 — (pdf 744 KB) [file 204_2024_3698_MOESM1_ESM.pdf]

Archives of Toxicology

# PBTK model-based analysis of CYP3A4 induction and the toxicokinetics of the pyrrolizidine alkaloid retrorsine in man

## Supplementary document

**Anja Lehmann[1,2] · Ina Geburek[1] · Stefanie Hessel-Pras[1] · Anne-Margarethe Enge[1] · Hans Mielke[1] · Christine Müller-Graf[1] · Charlotte Kloft[2] · Christoph Hethey[1]**

Corresponding author: Hans Mielke
Tel.: +49-30-18412-23303
E-mail: hans.mielke@bfr.bund.de

[1] German Federal Institute for Risk Assessment (BfR), Max-Dohrn-Str. 8-10, 10589 Berlin, Germany

[2] Department of Clinical Pharmacy and Biochemistry, Institute of Pharmacy, Freie Universitaet Berlin, 12169 Berlin, Germany

**Ordinary differential equations of the retrorsine-rifampicin interaction model**

The following ordinary differential equations describe the change of amount of substance (mol) of retrorsine, rifampicin or CYP3A4 over time t (h). All model parameters are listed in Tables S1 and S2.

Retrorsine in gut lumen (for oral administration):

$$\frac{d\mathrm{RET}_{\mathrm{lum}}(t)}{dt} = -k_{\mathrm{a}} \cdot F_{\mathrm{a}} \cdot \mathrm{RET}_{\mathrm{lum}}(t) \tag{S1}$$

Retrorsine in venous blood:

$$\frac{d\mathrm{RET}_{\mathrm{ven}}(t)}{dt} = \sum_{\mathrm{tis}} Q_{\mathrm{tis}} \cdot \frac{\mathrm{RET}_{\mathrm{tis}}(t)}{V_{\mathrm{tis}} \cdot K_{\mathrm{tis}}} + Q_{\mathrm{liv}} \cdot K_{\mathrm{liv}}^{\mathrm{vas:vi}} \cdot \frac{\mathrm{RET}_{\mathrm{liv}}^{\mathrm{vi}}(t)}{V_{\mathrm{liv}}^{\mathrm{vi}}} - Q_c \cdot \frac{\mathrm{RET}_{\mathrm{ven}}(t)}{V_{\mathrm{ven}}} \tag{S2}$$

$$\mathrm{tis} = \mathrm{adi}, \mathrm{bon}, \mathrm{bra}, \mathrm{hea}, \mathrm{kid}, \mathrm{mus}, \mathrm{ski}$$

Retrorsine in arterial blood:

$$\frac{d\mathrm{RET}_{\mathrm{art}}(t)}{dt} = Q_c \cdot \left(\frac{\mathrm{RET}_{\mathrm{lun}}(t)}{V_{\mathrm{lun}} \cdot K_{\mathrm{lun}}} - \frac{\mathrm{RET}_{\mathrm{art}}(t)}{V_{\mathrm{art}}}\right) \tag{S3}$$

Retrorsine in lungs:

$$\frac{d\mathrm{RET}_{\mathrm{lun}}(t)}{dt} = Q_c \cdot \left(\frac{\mathrm{RET}_{\mathrm{ven}}(t)}{V_{\mathrm{ven}}} - \frac{\mathrm{RET}_{\mathrm{lun}}(t)}{V_{\mathrm{lun}} \cdot K_{\mathrm{lun}}}\right) \tag{S4}$$

Retrorsine in adipose, bone, brain, heart, muscle, skin or spleen:

$$\frac{d\mathrm{RET}_{\mathrm{tis}}(t)}{dt} = Q_{\mathrm{tis}} \cdot \left(\frac{\mathrm{RET}_{\mathrm{art}}(t)}{V_{\mathrm{art}}} - \frac{\mathrm{RET}_{\mathrm{tis}}(t)}{V_{\mathrm{tis}} \cdot K_{\mathrm{tis}}}\right) \tag{S5}$$

$$\mathrm{tis} = \mathrm{adi}, \mathrm{bon}, \mathrm{bra}, \mathrm{hea}, \mathrm{mus}, \mathrm{ski}, \mathrm{spl}$$

Retrorsine in kidneys:

$$\frac{d\mathrm{RET}_{\mathrm{kid}}(t)}{dt} = Q_{\mathrm{kid}} \cdot \left(\frac{\mathrm{RET}_{\mathrm{art}}(t)}{V_{\mathrm{art}}} - \frac{\mathrm{RET}_{\mathrm{kid}}(t)}{V_{\mathrm{kid}} \cdot K_{\mathrm{kid}}}\right) - f_{\mathrm{u,p}} \cdot \mathrm{GFR} \cdot \frac{\mathrm{RET}_{\mathrm{kid}}(t)}{V_{\mathrm{kid}}} \tag{S6}$$

Retrorsine in urine:

$$\frac{d\mathrm{RET}_{\mathrm{uri}}(t)}{dt} = f_{\mathrm{u,p}} \cdot \mathrm{GFR} \cdot \frac{\mathrm{RET}_{\mathrm{kid}}(t)}{V_{\mathrm{kid}}} \tag{S7}$$

Retrorsine in in gut tissue:

$$\begin{aligned}\frac{d\mathrm{RET}_{\mathrm{gut}}(t)}{dt} &= k_{\mathrm{a}} \cdot F_{\mathrm{a}} \cdot \mathrm{RET}_{\mathrm{lum}}(t) + Q_{\mathrm{gut}} \cdot \left(\frac{\mathrm{RET}_{\mathrm{art}}(t)}{V_{\mathrm{art}}} - \frac{\mathrm{RET}_{\mathrm{gut}}(t)}{V_{\mathrm{gut}} \cdot K_{\mathrm{gut}}}\right) \\ &- F_{\mathrm{si}} \cdot (1 - f_{m,\mathrm{CYP3A4}}) \cdot \frac{V_{\mathrm{max,gut}}}{K_{\mathrm{M,gut}} + f_{\mathrm{u,gut}} \cdot \frac{\mathrm{RET}_{\mathrm{gut}}(t)}{V_{\mathrm{gut}}}} \cdot f_{\mathrm{u,gut}} \cdot \frac{\mathrm{RET}_{\mathrm{gut}}(t)}{V_{\mathrm{gut}}} \\ &- F_{\mathrm{si}} \cdot f_{m,\mathrm{CYP3A4}} \cdot \frac{k_{\mathrm{cat,gut}} \cdot \mathrm{CYP3A4}_{\mathrm{gut}}(t)}{K_{\mathrm{M,gut}} \cdot \left(1 + \underbrace{\frac{\mathrm{RIF}_{\mathrm{gut}}(t)}{V_{\mathrm{gut}}} \cdot \frac{1}{K_i}}_{\text{Competitive inhibition}}\right) + f_{\mathrm{u,gut}} \cdot \frac{\mathrm{RET}_{\mathrm{gut}}(t)}{V_{\mathrm{gut}}}} \cdot f_{\mathrm{u,gut}} \cdot \frac{\mathrm{RET}_{\mathrm{gut}}(t)}{V_{\mathrm{gut}}}\end{aligned} \tag{S8}$$

Retrorsine in liver vascular/interstitial (superscript vi, also termed extracellular) space:

$$\begin{aligned}\frac{d\mathrm{RET}^{\mathrm{vi}}_{\mathrm{liv}}(t)}{dt} &= (Q_{\mathrm{liv}} - Q_{\mathrm{spl}} - Q_{\mathrm{gut}}) \cdot \frac{\mathrm{RET}_{\mathrm{art}}(t)}{V_{\mathrm{art}}} + Q_{\mathrm{spl}} \cdot \frac{\mathrm{RET}_{\mathrm{spl}}(t)}{V_{\mathrm{spl}} \cdot K_{\mathrm{spl}}} \\ &+ Q_{\mathrm{gut}} \cdot \frac{\mathrm{RET}_{\mathrm{gut}}(t)}{V_{\mathrm{gut}} \cdot K_{\mathrm{gut}}} - Q_{\mathrm{liv}} \cdot K^{\mathrm{vas:vi}}_{\mathrm{liv}} \cdot \frac{\mathrm{RET}^{\mathrm{vi}}_{\mathrm{liv}}}{V^{\mathrm{vi}}_{\mathrm{liv}}} - (\mathrm{CL}_{\mathrm{act,in}} + \mathrm{PS}_{\mathrm{diff}}) \cdot K^{\mathrm{int,u:vi}}_{\mathrm{liv}} \cdot \frac{\mathrm{RET}^{\mathrm{vi}}_{\mathrm{liv}}(t)}{V^{\mathrm{vi}}_{\mathrm{liv}}} \\ &+ \mathrm{CL}_{\mathrm{act,ef}} \cdot f^{\mathrm{c}}_{\mathrm{u,liv}} \cdot \frac{\mathrm{RET}^{\mathrm{c}}_{\mathrm{liv}}(t)}{V^{\mathrm{c}}_{\mathrm{liv}}} + \mathrm{PS}_{\mathrm{diff}} \cdot \frac{f^{\mathrm{c}}_{\mathrm{n,liv}}}{f^{\mathrm{int}}_{\mathrm{n,liv}}} \cdot f^{\mathrm{c}}_{\mathrm{u,liv}} \cdot \frac{\mathrm{RET}^{\mathrm{c}}_{\mathrm{liv}}(t)}{V^{\mathrm{c}}_{\mathrm{liv}}} \\ &\text{with } K^{\mathrm{vas:vi}}_{\mathrm{liv}} = \frac{\mathrm{BP}}{f_{\mathrm{u,p}}} \cdot \left(\frac{V^{\mathrm{vas}}_{\mathrm{liv}}}{V^{\mathrm{vi}}_{\mathrm{liv}}} \cdot \frac{\mathrm{BP}}{f_{\mathrm{u,p}}} + \frac{V^{\mathrm{int}}_{\mathrm{liv}}}{V^{\mathrm{vi}}_{\mathrm{liv}}} \cdot \frac{1}{f^{\mathrm{int}}_{\mathrm{u,liv}}}\right)^{-1}, \\ &K^{\mathrm{int,u:vi}}_{\mathrm{liv}} = f^{\mathrm{int}}_{\mathrm{u,liv}} \cdot \left(\frac{V^{\mathrm{vas}}_{\mathrm{liv}}}{V^{\mathrm{vi}}_{\mathrm{liv}}} \cdot \frac{\mathrm{BP}}{f_{\mathrm{u,p}}} + \frac{V^{\mathrm{int}}_{\mathrm{liv}}}{V^{\mathrm{vi}}_{\mathrm{liv}}} \cdot \frac{1}{f^{\mathrm{int}}_{\mathrm{u,liv}}}\right)^{-1}\end{aligned} \tag{S9}$$

Retrorsine in liver cellular (superscript c) space:

$$\begin{aligned}\frac{d\mathrm{RET}^{\mathrm{c}}_{\mathrm{liv}}(t)}{dt} &= (\mathrm{CL}_{\mathrm{act,in}} + \mathrm{PS}_{\mathrm{diff}}) \cdot K^{\mathrm{int,u:vi}}_{\mathrm{liv}} \cdot \frac{\mathrm{RET}^{\mathrm{vi}}_{\mathrm{liv}}(t)}{V^{\mathrm{vi}}_{\mathrm{liv}}} - \mathrm{CL}_{\mathrm{act,ef}} \cdot f^{\mathrm{c}}_{\mathrm{u,liv}} \frac{\mathrm{RET}^{\mathrm{c}}_{\mathrm{liv}}(t)}{V^{\mathrm{c}}_{\mathrm{liv}}} \\ &- \mathrm{PS}_{\mathrm{diff}} \frac{f^{\mathrm{c}}_{\mathrm{n,liv}}}{f^{\mathrm{int}}_{\mathrm{n,liv}}} \cdot f^{\mathrm{c}}_{\mathrm{u,liv}} \frac{\mathrm{RET}^{\mathrm{c}}_{\mathrm{liv}}(t)}{V^{\mathrm{c}}_{\mathrm{liv}}} - \mathrm{CL}_{\mathrm{bile}} \cdot f^{\mathrm{c}}_{\mathrm{u,liv}} \frac{\mathrm{RET}^{\mathrm{c}}_{\mathrm{liv}}(t)}{V^{\mathrm{c}}_{\mathrm{liv}}} \\ &- (1 - f_{\mathrm{m,CYP3A4}}) \cdot \frac{V_{\mathrm{max,liv}}}{K_{\mathrm{M,liv}} + f^{\mathrm{c}}_{\mathrm{u,liv}} \frac{\mathrm{RET}^{\mathrm{c}}_{\mathrm{liv}}(t)}{V^{\mathrm{c}}_{\mathrm{liv}}}} \cdot f^{\mathrm{c}}_{\mathrm{u,liv}} \frac{\mathrm{RET}^{\mathrm{c}}_{\mathrm{liv}}(t)}{V^{\mathrm{c}}_{\mathrm{liv}}} \\ &- f_{\mathrm{m,CYP3A4}} \cdot \frac{k_{\mathrm{cat,liv}} \cdot \mathrm{CYP3A4}^{\mathrm{c}}_{\mathrm{liv}}(t)}{K_{\mathrm{M,liv}} \cdot \left(1 + \underbrace{\frac{\mathrm{RIF}^{\mathrm{c}}_{\mathrm{liv}}(t)}{V^{c}_{\mathrm{liv}}} \cdot \frac{1}{K_i}}_{\text{Competitive inhibition}}\right) + f^{\mathrm{c}}_{\mathrm{u,liv}} \cdot \frac{\mathrm{RET}^{\mathrm{c}}_{\mathrm{liv}}(t)}{V^{\mathrm{c}}_{\mathrm{liv}}}} \cdot f^{\mathrm{c}}_{\mathrm{u,liv}} \cdot \frac{\mathrm{RET}^{\mathrm{c}}_{\mathrm{liv}}(t)}{V^{\mathrm{c}}_{\mathrm{liv}}}\end{aligned} \tag{S10}$$

CYP3A4 in liver cellular space:

$$\frac{dCYP3A4^{c}_{liv}(t)}{dt} = k_{deg,liv} \cdot CYP3A4^{c}_{0,liv} \cdot \Bigg(1 + \underbrace{\frac{E_{max} \cdot \frac{RIF^{c}_{liv}(t)}{V liv^{c}}}{EC_{50} + \frac{RIF^{c}_{liv}(t)}{V^{c}_{liv}}}}_{\text{Induction}}\Bigg) - k_{deg,liv} \cdot CYP3A4^{c}_{liv}(t) \tag{S11}$$

CYP3A4 in gut tissue:

$$\frac{dCYP3A4_{gut}(t)}{dt} = k_{deg,gut} \cdot CYP3A4_{0,gut} \cdot \Bigg(1 + \underbrace{\frac{E_{max} \cdot \frac{RIF_{gut}(t)}{V gut}}{EC_{50} + \frac{RIF_{gut}(t)}{V_{gut}}}}_{\text{Induction}}\Bigg) - k_{deg,gut} \cdot CYP3A4_{gut}(t) \tag{S12}$$

The following two-compartment model with first order-absorption and elimination was used to describe rifampicin concentrations in the liver cellular space (liv,c) as well as in the small intestine cellular space (gut). The distribution from the central compartment to the peripheral compartment and vice versa was described by the transition rate constants $k_{12,tis}$ and $k_{k21,tis}$. First-order absorption from a depot compartment was parametrized by the absorption rate constant $k_{depot,tis}$. Elimination of rifampicin from the central compartment was described as first-order process with the elimination rate constant $k_{e,tis}$.

Rifampicin in the depot compartment:

$$\begin{aligned} \frac{dRIF_{depot,tis}(t)}{dt} &= -k_{depot,tis} \cdot RIF_{depot,tis}(t) \\ tis &= \text{liv,c, gut} \end{aligned} \tag{S13}$$

Rifampicin in the central compartment:

$$\begin{aligned} \frac{dRIF_{cen,tis}(t)}{dt} &= k_{depot,tis} \cdot RIF_{depot,tis}(t) + k_{21,tis} \cdot RIF_{per,tis} \\ &\quad - k_{12,tis} \cdot RIF_{cen,tis} - k_{e,tis} \cdot RIF_{cen,tis} \\ tis &= \text{liv,c, gut} \end{aligned} \tag{S14}$$

Rifampicin in the peripheral compartment:

$$\begin{aligned} \frac{dRIF_{per,tis}(t)}{dt} &= k_{12,tis} \cdot RIF_{cen,tis} - k_{21,tis} \cdot RIF_{per,tis} \\ tis &= \text{liv,c, gut} \end{aligned} \tag{S15}$$

**Table S1** Final parameters of the PBTK model for retrorsine in man

| Parameter | Unit | Additional description | Value | Source |
|---|---|---|---|---|
| **Body weight**† | | | | |
| bw | kg | | 73 | ICRP (2002) |
| **Tissue volumes**† | | | | |
| $V_{blo}$ | L | Blood | 3.42 | ICRP (2002) |
| $V_{ven}$ | L | Venous blood | 2.41 | Assumed 2/3 of $V_{blo}$ |
| $V_{art}$ | L | Arterial blood | 1.01 | Assumed 1/3 of $V_{blo}$ |
| $V_{adi}$ | L | Adipose | 15.8 | ICRP (2002) |
| $V_{bon}$ | L | Bone | 8.08 | ICRP (2002) |
| $V_{bra}$ | L | Brain | 1.45 | ICRP (2002) |
| $V_{gut}$ | L | Gut tissue | 1.02 | ICRP (2002) |
| $V_{hea}$ | L | Heart | 0.330 | ICRP (2002) |
| $V_{kid}$ | L | Kidneys | 0.310 | ICRP (2002) |
| $V_{liv}$ | L | Liver | 1.80 | ICRP (2002) |
| $V_{liv}^{c}$ | L | Liver cellular space | 1.30 | ICRP (2002); Kawai et al. (1994) |
| $V_{liv}^{vi}$ | L | Liver vascular/interstitial space | 0.500 | ICRP (2002); Kawai et al. (1994) |
| $V_{lun}$ | L | Lungs | 0.500 | ICRP (2002) |
| $V_{mus}$ | L | Muscle | 29.0 | ICRP (2002) |
| $V_{ski}$ | L | Skin | 3.30 | ICRP (2002) |
| $V_{spl}$ | L | Spleen | 0.150 | ICRP (2002) |
| **Tissue blood flows**† | | | | |
| $Q_{adi}$ | L/h | Adipose | 19.5 | ICRP (2002) |
| $Q_{bon}$ | L/h | Bone | 19.5 | ICRP (2002) |
| $Q_{bra}$ | L/h | Brain | 46.8 | ICRP (2002) |
| $Q_{gut}$ | L/h | Gut tissue | 54.6 | ICRP (2002) |
| $Q_{hea}$ | L/h | Heart | 15.6 | ICRP (2002) |
| $Q_{kid}$ | L/h | Kidneys | 74.1 | ICRP (2002) |
| $Q_{liv}$ | L/h | Liver | 99.5 | ICRP (2002) |
| $Q_{mus}$ | L/h | Muscle | 66.3 | ICRP (2002) |
| $Q_{ski}$ | L/h | Skin | 19.5 | ICRP (2002) |
| $Q_{spl}$ | L/h | Spleen | 11.7 | ICRP (2002) |
| **Cardiac output**† | | | | |
| $Q_{c}$ | L/h | | 360.8 | ICRP (2002) |
| **Glomerular filtration rate**† | | | | |
| GFR | L/h | | 6.57 | Benjamin et al. (2015) |
| **Hematocrit** | | | | |
| hct | fraction | Fraction of red blood cells in blood | 0.4 | Baskurt and Windberger (2007) |
| **Physico-/ biochemical properties of retrorsine** | | | | |
| subclass | - | Neutral/acid/weak base/string base | weak base | |
| $pK_a$ | - | Acid strength | 6.86 | Predicted using the web application SPARC (SPARC 2019) |
| $\log P$ | - | Lipophilicity | -1.26 | Haas et al. (2019) |
| BP | - | Blood-to-plasma ratio | 1.08 | Assumed equal to human BP, Haas et al. (2019) |
| $f_{u,p}$ | fraction | Fraction unbound in plasma | 0.600 | Assumed equal to human $f_{u,p}$ , Haas et al. (2019) |
| $f_{u,liv}^{int}$ | fraction | Fraction unbound in liver interstitium | 0.600 | Assumed equal to $f_{u,p}$ |
| $f_{u,liv}^{c}$ | fraction | Fraction unbound in liver cell | 0.600 | Assumed equal to $f_{u,p}$ |
| $f_{u,gut}$ | fraction | Fraction unbound in gut tissue | 0.600 | Assumed equal to $f_{u,p}$ |
| $f_{n,liv}^{int}$ | fraction | Fraction neutral in liver interstitium | 0.776 | Henderson-Hasselbalch equation |
| $f_{n,liv}^{c}$ | fraction | Fraction neutral in liver cell | 0.701 | Henderson-Hasselbalch equation |
| **Tissue-to-plasma partition coefficients of retrorsine**† | | | | |
| $K_{adi}$ | - | Adipose | 0.222 | Predicted as in Rodgers and Rowland (2006) |
| $K_{bon}$ | - | Bone | 0.625 | Predicted as in Rodgers and Rowland (2006) |
| $K_{bra}$ | - | Brain | 1.04 | Predicted as in Rodgers and Rowland (2006) |
| $K_{gut}$ | - | Gut tissue | 0.987 | Predicted as in Rodgers and Rowland (2006) |
| $K_{hea}$ | - | Heart | 1.02 | Predicted as in Rodgers and Rowland (2006) |
| $K_{kid}$ | - | Kidneys | 1.05 | Predicted as in Rodgers and Rowland (2006) |
| $K_{liv}^{vas:vi}$ | - | Liver vascular-to-lumped compartment partition coefficient | 1.05 | Predicted as in Schweinoch (2014), Eq.S9 |
| $K_{liv}^{int,u:vi}$ | - | Liver unbound interstitial-to-lumped compartment partition coefficient | 0.348 | Predicted as in Schweinoch (2014), Eq.S9 |

Continued on next page

**Table S1** Continued from previous page

| Parameter | Unit | Additional description | Value | Source |
|---|---|---|---|---|
| $K_{lun}$ | - | Lungs | 1.11 | Predicted as in Rodgers and Rowland (2006) |
| $K_{mus}$ | - | Muscle | 1.02 | Predicted as in Rodgers and Rowland (2006) |
| $K_{ski}$ | - | Skin | 1.01 | Predicted as in Rodgers and Rowland (2006) |
| $K_{spl}$ | - | Spleen | 1.05 | Predicted as in Rodgers and Rowland (2006) |
| **Liver transport of retrorsine** | | | | |
| $PS_{diff}$ | mL/min/g liver | Passive influx diffusion flow rate | 0.124 | Medium loss assay |
| $CL_{act,in}$ | mL/min/g liver | Active uptake clearance | 1.02 | Medium loss assay |
| $CL_{act,ef}$ | mL/min/g liver | Active efflux clearance | 0 | Assumed negligible |
| $CL_{bile}$ | mL/min/g liver | Biliary excretion clearance | 0 | Assumed negligible |
| **Liver metabolism of retrorsine** | | | | |
| $K_{M,liv}$ | $\mu$M | Concentration at half-maximal metabolism reaction velocity | 25.5 | Liver microsomal assay |
| $V_{max,liv}$ | nmol/min/g liver | Maximum metabolism reaction velocity | 26.2 | Liver microsomal assay |
| $k_{catCYP3A,liv}$ | 1/min | CYP3A4 catalytic constant | 5.59 | Assumed equal to $V_{max,liv}/CYP3A4^{c}_{0,liv}$ |
| $f_{m,CYP3A4}$ | fraction | Fraction metabolized by CYP3A4 | 0.527 | Derived from Ruan et al. (2014) |
| **Intestinal metabolism of retrorsine** | | | | |
| $F_{si}$ | fraction | Volume fraction of gut tissue that is intracellular of small intestine | 0.562 | Derived from Hanke et al. (2018) |
| $K_{M,gut}$ | $\mu$M | Concentration at half-maximal metabolism reaction velocity | 25.5 | Assumed equal to $K_{M,liv}$ |
| $V_{max,gut}$ | nmol/min/g gut | Maximum metabolism reaction velocity | 2.62 | Assumed $\frac{1}{10} \cdot V_{max,liv}$ |
| $k_{catCYP3A,gut}$ | 1/min | CYP3A4 catalytic constant | 13.0 | Assumed equal to $V_{max,gut}/CYP3A4^{c}_{0,gut}$ |
| **Intestinal absorption of retrorsine** | | | | |
| $F_a$ | fraction | Intestinal fraction absorbed | 0.783 | Lehmann et al. (2023) |
| $k_a$ | 1/h | Intestinal absorption rate constant | 0.910 | Lehmann et al. (2023) |
| **CYP3A4 enzyme properties** | | | | |
| $k_{deg}$ | 1/h | Degradation rate contant | 0.0193 | $t_{1/2}$= 36 h; Hanke et al. (2018); Rowland Yeo et al. (2011) |
| $CYP3A4^{c}_{0,liv}$ | µmol | CYP3A baseline amount in liver cellular space | 8.42 | derived from $CYP3A4_{0,liv}$ conc. reported in Hanke et al. (2018) |
| $CYP3A4_{0,gut}$ | µmol | CYP3A baseline amount in gut tissue | 0.363 | derived from $CYP3A4_{0,gut}$ conc. reported in Hanke et al. (2018) |
| **CYP3A4 induction by rifampicin** | | | | |
| $EC_{50}$ | µmol/L | Concentration at half-maximal induction in vivo | 0.34 | Hanke et al. (2018); Shou et al. (2008); Templeton et al. (2011) |
| $E_{max}$ | - | Maximum induction effect in vivo | 9.00 | Hanke et al. (2018); Templeton et al. (2011) |
| **CYP3A4 competitive inhibition by rifampicin** | | | | |
| $K_i$ | µmol/L | Concentration for half-maximal inhibition | 18.5 | Hanke et al. (2018); Kajosaari et al. (2005) |

† Physiological parameters and prediction of tissue-to-plasma partition coefficients were based on the MATLAB-based pharmacometric modeling framework developed by Hartung and Huisinga (2019)

**Table S2** Parameter estimates of the two compartment model that was used to describe PBPK model-based rifampicin concentrations in the liver cellular space and the gut tissue

| Parameter | Unit | Additional Description | Value | 95% Credible interval |
|---|---|---|---|---|
| **Liver cellular space** | | | | |
| $k_{depot,liv,c}$ | 1/h | Absorption rate constant | 0.357 | [0.352, 0.361] |
| $k_{12,liv,c}$ | 1/h | Transition rate constant 12 | 0.279 | [0.269, 0.288] |
| $k_{21,liv,c}$ | 1/h | Transition rate constant 21 | 0.155 | [0.146, 0.164] |
| $k_{e,liv,c}$ | 1/h | Elimination rate constant | 0.548 | [0.545, 0.551] |
| **Gut tissue** | | | | |
| $k_{depot,gut}$ | 1/h | Absorption rate constant | 0.187 | [0.182, 0.192] |
| $k_{12,gut}$ | 1/h | Transition rate constant 12 | 0.716 | [0.620, 0.796] |
| $k_{21,gut}$ | 1/h | Transition rate constant 21 | 0.0258 | [0.0224, 0.0298] |
| $k_{e,gut}$ | 1/h | Elimination rate constant | 5.77 | [5.68, 5.84] |

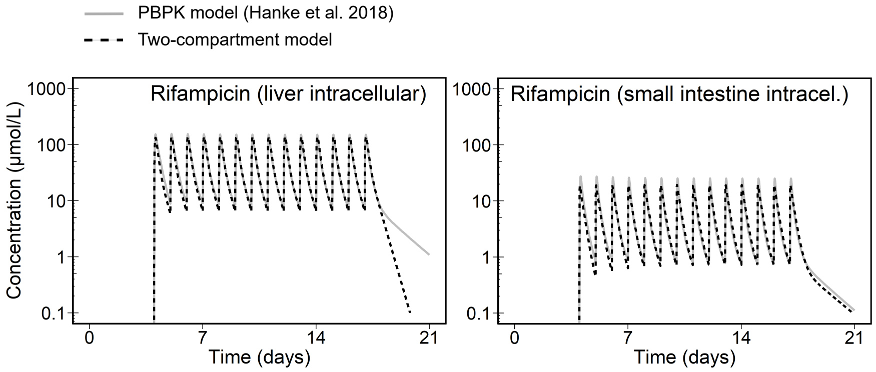


**Fig. S1** Rifampicin concentration-time profiles after 600 mg q.d. rifampicin treatment

## Evaluation of the 2-compartment model of rifampicin

A 2-compartment model was set up and calibrated with PBPK model predictions (Hanke et al. 2018) of intracellular rifampicin concentrations of the liver and of the small intestine, separately. In both cases, all parameters were identifiable and estimated with low uncertainty (Table S2). Rifampicin concentrations in both tissues were well described by the 2-compartment model as shown in Fig. S1.

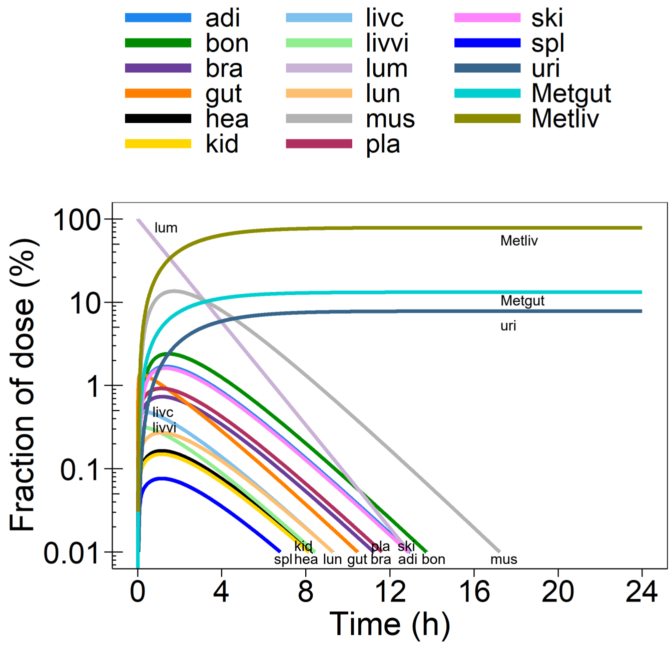


**Fig. S2** Predicted disposition pattern (fraction of administered dose (%)) after single oral dose of 1 µg/kg body weight retrorsine. **Abbreviations:** adi adipose; bon bone; bra brain; gut gut tissue; hea heart; kid kidneys; livc liver cellular space; livvi liver vascular/interstitial space; lum gut lumen; lun lungs; mus muscle; pla plasma; ski skin; spl spleen; uri urine; Metgut total gut metabolites; Metliv total liver metabolites. **Note:** uri, Metgut and Metliv are given as cumulative fraction of dose (%)

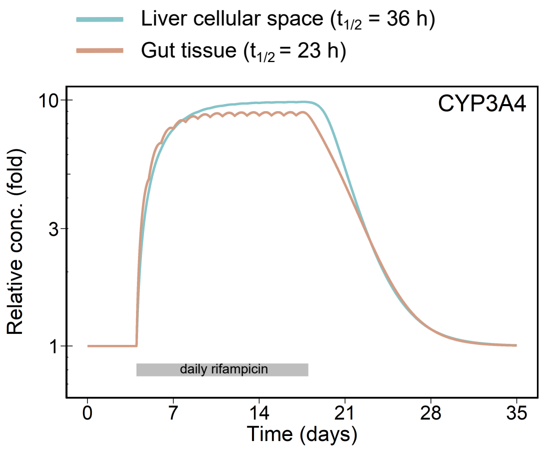


**Fig. S3** Fold change of predicted CYP3A4 concentration in the liver cellular space and in the gut as a result of daily oral intake of 600 mg rifampicin (day 4 to 18)

## Chemicals and biological materials

Retrorsine was purchased from AppliChem (Darmstadt, Germany) and from Phytoplan (Heidelberg, Germany). William's Medium E and supplements were purchased from PAN-Biotech (Aidenbach, Germany), Sigma-Aldrich (Taufkirchen, Germany) and Capricorn Scientific (Ebsdorfergrund, Germany). Methanol and water (LC–MS grade) were obtained from Merck KGA (Darmstadt, Germany). All chemicals and co-factors for liver microsomal assays were purchased from Carl Roth (Karlsruhe, Germany) or Sigma-Aldrich (Steinheim, Germany). Human liver microsomes were obtained by BD Biosciences (Woburn, MA, USA). Characteristics of microsomal preparations are summarized in Table S3. The human hepatoma cell line HepaRG was obtained from Biopredic International (Saint-Grégoire, France).

## Culture of HepaRG cells

The culture conditions of the human hepatoma cell line HepaRG were previously published in Enge et al. (2021) (Section 4.2. Cell Culture). Briefly, cells were seeded and cultivated in William's Medium E with stable glutamine supplemented with 10% fetal bovine serum, 5 µg/mL human insulin, 50 µM hydrocortisone hemisuccinate, 100 U/mL penicillin and 100 µg/mL streptomycin at 37 °C in an atmosphere of 5% CO2. After two weeks of proliferation, differentiation was initiated by adding 1.7% of dimethyl sulfoxide. After additional two weeks of cultivation, HepaRG cells were fully differentiated. All experiments were performed with differentiated cells seeded at passages 16 to 20.
To measure time-dependent depletion of retrorsine in cell culture medium, HepaRG cells were seeded at a density of $0.2 \cdot 10^6$ cells per well in a 6-well plate and cultivated as described above. HepaRG cells were adapted to serum-free medium and medium substituted with insulin, transferrin, selenium (ITS) serum extender 48 h prior to retrorsine incubation for optimal LC-MS/MS measurement.

**Table S3** Characteristics of the human liver microsomal pool as provided by the manufacturer

| Characteristic | Unit | Value |
|---|---|---|
| No. of donors | - | 50 (25 males, 25 females) |
| Age span | years | 21 to 77 |
| Race | - | Caucasian (45), Hispanic (2), Asian (1), African American (1), unknown (1) |
| Protein concentration | mg/mL | 20 |
| CYP concentration | pmol/mg prot | |
| Total CYP | | 310 |
| CYP3A4 | | 86 |
| CYP3A5 | | 9.8 |
| CYP activity | pmol/mg/min | |
| CYP1A2 | | 770 |
| CYP2A6 | | 1100 |
| CYP2B6 | | 59 |
| CYP2C8 | | 110 |
| CYP2C9 | | 2700 |
| CYP2C19 | | 70 |
| CYP2D6 | | 61 |
| CYP2E1 | | 2800 |
| CYP3A4 | | 5300 |
| CYP4A11 | | 1300 |
| FMO | | 560 |
| UGT1A1 | | 820 |
| UGT1A4 | | 650 |
| UGT1A6 | | 11000 |
| UGT1A9 | | 2300 |
| UGT2B7 | | 620 |

## Medium loss assay

Measurement of time-dependent retrorsine depletion in HepaRG cells was previously published in Enge et al. (2021) (Section 4.5. Transport Analysis of PAs in HepaRG Cells). At the start of the experiment, cells were treated with 0.7 µM retrorsine at 4°C and 37°C. Cell culture medium samples were taken at 0, 120 and 240 min. Samples were stored at -20°C until they were diluted in 5% methanol and analyzed for retrorsine by LC-MS/MS as reported in Enge et al. (2021) (Section 4.6. Analysis of PA and PANO Content in Cell Culture Supernatant Using LC-MS/MS).
Measured retrorsine concentrations were normalized to the initial retrorsine concentration $RET_0$ for each biological replicate. Retrorsine depletion-time profiles were described by a monoexponential model, where the parameter $\lambda_T$ (1/h) represents the rate constant of retrorsine loss in medium at the respective temperature T:

$$\frac{\mathrm{RET}(t)}{\mathrm{RET}_0} = e^{-\lambda_T \cdot t}$$
$$\mathrm{T}(^\circ\mathrm{C}) = 4, 37 \quad \text{(S16)}$$

A 2-compartment model (Fig. S4) was used to derive the passive influx diffusion flow rate into the cells $PS_{\mathrm{diff,in}\,\mathit{in\,vitro}}$ and the active uptake clearance $CL_{\mathrm{act,in}\,\mathit{in\,vitro}}$ (both L/h/$10^6$ cells). $PS_{\mathrm{diff,in}\,\mathit{in\,vitro}}$ and $CL_{\mathrm{act,in}\,\mathit{in\,vitro}}$ (both L/h/$10^6$ cells) were approximated under the assumption that passive diffusion out of the cells is negligible during the first monoexponential

phase (Eqs. S17-S18). In vitro-to-in vivo extrapolation of both parameters was performed by multiplication with hepatocellularity $SF_{\mathrm{liv}}$ yielding in vivo $PS_{\mathrm{diff,in}}$ and $CL_{\mathrm{act,in}}$ (both L/h/g liver) (Eqs. S19 -S20).

$$PS_{\mathrm{diff,in}\,in\,vitro} = \lambda_{4^{\circ}\mathrm{C}} \cdot \frac{1}{C_{\mathrm{cell}}} \tag{S17}$$

$$CL_{\mathrm{act,in}\,in\,vitro} = \lambda_{37^{\circ}\mathrm{C}} \cdot \frac{1}{C_{\mathrm{cell}}} - PS_{\mathrm{diff,in}\,in\,vitro} \tag{S18}$$

$$PS_{\mathrm{diff,in}} = PS_{\mathrm{diff,in}\,in\,vitro} \cdot SF_{\mathrm{liv}} \cdot f_{\mathrm{u},\,in\,vitro} \tag{S19}$$

$$CL_{\mathrm{act,in}} = CL_{\mathrm{act,in}\,in\,vitro} \cdot SF_{\mathrm{liv}} \cdot f_{\mathrm{u},\,in\,vitro} \tag{S20}$$

where:

| | | | |
|---|---|---|---|
| $C_{\mathrm{cell}}$ | Cell density (cells/mL): | $1.00 \cdot 10^5$ | (Experimental) |
| $f_{\mathrm{u},\,in\,vitro}$ | Fraction unbound in vitro | 1.00 | (Assumption) |
| $SF_{\mathrm{liv}}$ | Hepatocellularity (cells/g liver): | $99 \cdot 10^6$ | (Ring et al. 2011) |

$f_{\mathrm{u},\,in\,vitro}$ was assumed 1, which was supported by the predicted value of 0.994 (Eq. 16 in Austin et al. (2005)). The predicted low protein binding is in line with the low lipophilicity of retrorsine (log$P$=-1.26).

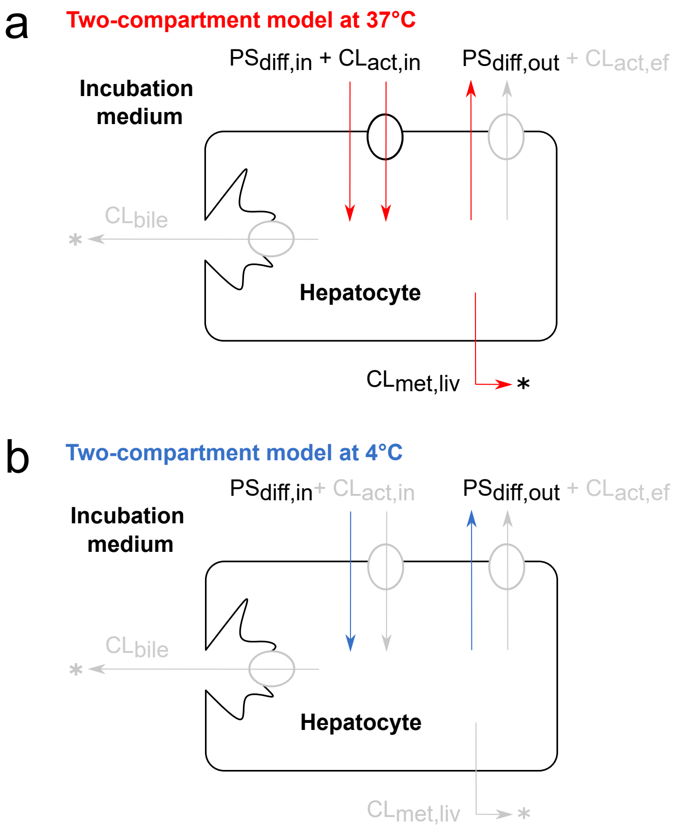


**Fig. S4** 2-Compartment model characterizing the uptake and clearance processes involved in the medium loss assay based on Schweinoch (2014). The external compartment represents the incubation medium surrounding the liver cell, while the internal compartment represents the intracellular space of the liver cell. It was assumed that in the medium loss assay most compound exporting proteins are not active in unpolarized monolayers of liver cells (absence of biliary clearance $CL_{bile}$ and active efflux $CL_{act,ef}$). At a physiological temperature of 37°C active influx $CL_{act,in}$, metabolism $CL_{met,liv}$ and passive diffusion $PS_{diff,in}$, $PS_{diff,out}$ were involved in compound clearance (**a**). At a non-physiological temperature of 4°C active influx and metabolism were regarded as negligible (**b**). Note: $PS_{diff,in}$ and $CL_{act,in}$ (Eqs. S17-S20) were approximated under the assumption that passive diffusion out of the cells $PS_{diff,out}$ is negligible during the first monoexponential phase

## Liver microsomal assay

Kinetics of retrorsine hepatic metabolism were determined by measuring retrorsine depletion in the presence of pooled human liver microsomes. Incubation mixtures were prepared on ice. Microsomal preparations were diluted in 50 mM Tris-HCl buffer (pH 7.5) to yield a protein concentration of 1 mg protein/mL. For simulation of phase I metabolism 33 mM potassium chloride, 8 mM magnesium chloride, 1 mM nicotinamide adenine dinucleotide phosphate (NADPH), 5 mM glucose-6-phosphate and 0.5 U/mL glucose-6-phosphate dehydrogenase were added. 2 mM glutathione were included to fasciliate formation of phase II glutathione conjugates. Mixtures were incubated at 37°C and 400 rpm with 1, 15, 50 and

200 μM of retrorsine. Reactions were stopped at 8, 15, 20, 30, 40, 50 and 60 min by addition of ice-cold methanol containing 1% ammonium formate. All experiments were performed in duplicate. Samples were vortexted and stored at -80°C. Thawed samples were centrifuged at 14,000xg at 4°C to precipitate salts and proteins. Supernatants were diluted in 5% methanol and were analyzed for retrorsinne by LC-MS/MS as previously reported in Geburek et al. (2020).

Measured retrorsine concentrations were normalized to the initial retrorsine concentration $\mathrm{RET}_0$. The following end-product inhibition model was developed to describe time-dependent retrorsine depletion:

$$\mathrm{RET} \xrightarrow{k} P \text{ with } P \text{ inhibiting } k \tag{S21}$$

$$\frac{d\mathrm{RET}(t)}{dt} = -k(P)\cdot \mathrm{RET}(t) \tag{S22}$$

$$\frac{dP(t)}{dt} = k(P)\cdot \mathrm{RET}(t) \tag{S23}$$

$$k(P) = k_0(\mathrm{RET}_0)\cdot\left(1-\frac{P(t)}{P(t)+\mathrm{IC}_{50}}\right) \tag{S24}$$

$$\text{with } k_0(\mathrm{RET}_0) = \frac{V_{\mathrm{max,liv},\mathit{in\,vitro}}}{K_{\mathrm{M,liv}}}\cdot\left(1-\frac{\mathrm{RET}_0}{\mathrm{RET}_0+K_{\mathrm{M,liv}}}\right)$$

In the end-product inhibition model, we assumed that RET ($\mu$M) is converted to a mixture of products $P$ with reaction rate constant $k$ (1/h) (Eqs. S21-S23). Highly reactive dehydroretrorsine (DHR), one of RET's reaction products, was considered to unspecifically bind to the active center of microsomal enzymes thereby inhibiting their activity over time. The irreversible inhibition of CYP3A4 by resulting from metabolic activation of RET has been demonstrated in vitro by Dai et al. (2010). As a consequence $k$ is inhibited in dependency of the product concentration. Half-maximal inhibition of $k$ is achieved at the product concentration $\mathrm{IC}_{50}$ ($\mu$M) (Eq. S24). $k_0$ represents the initial reaction rate constant where inhibition is still absent ($t = 0$). Its dependency on $\mathrm{RET}_0$ was described by a Michaelis-Menten-like relationship (Obach and Reed-Hagen 2002) with maximum reaction velocity $V_{\mathrm{max,liv},\mathit{in\,vitro}}$ ($\mu$M/min) and RET concentration at half-maximal reaction velocity $K_\mathrm{M}$ ($\mu$M). In vitro-to-in vivo extrapolation of $V_{\mathrm{max,liv},\mathit{in\,vitro}}$ yielded $V_{\mathrm{max,liv}}$ (nmol/min/g liver):

$$V_{\mathrm{max,liv}} = V_{\mathrm{max,liv},\mathit{in\,vitro}}\cdot\frac{1}{\rho_{\mathrm{prot}}}\cdot \mathrm{SF}_{\mathrm{microsomes}}\cdot f_{\mathrm{u},\,\mathit{in\ vitro}} \tag{S25}$$

where:

| | | | |
|---|---|---|---|
| $\rho_{\mathrm{prot}}$ | Protein concentration (mg protein/mL): | 1.00 | (Experimental) |
| $f_{\mathrm{u},\,\mathit{in\ vitro}}$ | Fraction unbound in vitro | 1.00 | (Assumption) |
| $\mathrm{SF}_{\mathrm{microsomes}}$ | Scaling factor (mg protein/g liver): | 32.0 | (Ring et al. 2011) |

$f_{\mathrm{u},\,\mathit{in\ vitro}}$ was assumed 1, which was supported by the predicted value of 0.998 (Eq. for bases in Turner et al. (2007)). The predicted low protein binding is in line with the low lipophilicity of retrorsine (log$P$=-1.26).

## References

Austin RP, Barton P, Mohmed S, Riley RJ (2005) The binding of drugs to hepatocytes and its relationship to physicochemical properties. Drug Metab Dispos 33(3):419–425, `https://doi.org/10.1124/dmd.104.002436`

Baskurt O, Windberger U (2007) Comparative Hemorheology. In: Baskurt OK, Hardeman, MR, Rampling MW, Meiselman H.J (Ed.) Handbook of Hemorheology and Hemodynamics, IOS Press, pp 267–288

Benjamin A, Gallacher DJ, Greiter-Wilke A, Guillon JM, Kasai C, Ledieu D, Levesque P, Prelle K, Ratcliffe S, Sannajust F, Valentin JP (2015) Renal studies in safety pharmacology and toxicology: A survey conducted in the top 15 pharmaceutical companies. Journal of Pharmacological and Toxicological Methods 75:101–110, https://doi.org/10.1016/j.vascn.2015.01.004

Dai J, Zhang F, Zheng J (2010) Retrorsine, but not monocrotaline, is a mechanism-based inactivator of P450 3A4. Chemico-Biological Interactions 183(1):49–56, http://doi.org/10.1016/j.cbi.2009.10.001

Enge AM, Kaltner F, Gottschalk C, Braeuning A, Hessel-Pras S (2021) Active Transport of Hepatotoxic Pyrrolizidine Alkaloids in HepaRG Cells. Int J Mol Sci 22(8):3821, https://doi.org/10.3390/ijms22083821

Geburek I, Preiss-Weigert A, Lahrssen-Wiederholt M, Schrenk D, These A (2020) In vitro metabolism of pyrrolizidine alkaloids – Metabolic degradation and GSH conjugate formation of different structure types. Food and Chemical Toxicology 135:110868, https://doi.org/10.1016/j.fct.2019.110868

Haas M, Tänzer J, Hamscher G, Lehmann A, Hethey C, These A (2019) Bestimmung von stoffabhängigen Parametern zur toxikokinetischen Modellierung von Pyrrolizidinalkaloiden. Lebensmittelchemie 73(S1):S142–S142, https://doi.org/10.1002/lemi.201951142

Hanke N, Frechen S, Moj D, Britz H, Eissing T, Wendl T, Lehr T (2018) PBPK Models for CYP3A4 and P-gp DDI Prediction: A Modeling Network of Rifampicin, Itraconazole, Clarithromycin, Midazolam, Alfentanil, and Digoxin. CPT Pharmacometrics Syst Pharmacol 7(10):647–659, https://doi.org/10.1002/psp4.12343

Hartung N, Huisinga W (2019) A flexible and transparent MATLAB framework for empirical and mechanistic pharmacometric modelling. www.page-meeting.org/?abstract=9082

ICRP (2002) Basic anatomical and physiological data for use in radiological protection: Reference values. A report of age- and gender-related differences in the anatomical and physiological characteristics of reference individuals. ICRP Publication 89. Ann ICRP 32(3-4):5–265, https://journals.sagepub.com/doi/pdf/10.1177/ANIB_32_3-4

Kajosaari LI, Laitila J, Neuvonen PJ, Backman JT (2005) Metabolism of repaglinide by CYP2C8 and CYP3A4 in vitro: Effect of fibrates and rifampicin. Basic Clin Pharmacol Toxicol 97(4):249–256, https://doi.org/10.1111/j.1742-7843.2005.pto_157.x

Kawai R, Lemaire M, Steimer JL, Bruelisauer A, Niederberger W, Rowland M (1994) Physiologically based pharmacokinetic study on a cyclosporin derivative, SDZ IMM 125. J Pharmacokinet Biopharm 22(5):327–365, https://doi.org/10.1007/BF02353860

Lehmann A, Geburek I, Hessel-Pras S, Hengstler JG, Albrecht W, Mielke H, Müller-Graf C, Yang X, Kloft C, Hethey C (2023) PBTK modeling of the pyrrolizidine alkaloid retrorsine to predict liver toxicity in mouse and rat. Arch Toxicol 97(5):1319–1333, DOI 10.1007/s00204-023-03453-z

Obach RS, Reed-Hagen AE (2002) Measurement of Michaelis constants for cytochrome P450-mediated biotransformation reactions using a substrate depletion approach. Drug Metab Dispos 30(7):831–837, https://doi.org/10.1124/dmd.30.7.831

Ring BJ, Chien JY, Adkison KK, Jones HM, Rowland M, Jones RD, Yates JWT, Ku MS, Gibson CR, He H, Vuppugalla R, Marathe P, Fischer V, Dutta S, Sinha VK, Björnsson T, Lavé T, Poulin P (2011) PhRMA CPCDC initiative on predictive models of human phar-

macokinetics, part 3: Comparative assessement of prediction methods of human clearance. J Pharm Sci 100(10):4090–4110, https://doi.org/10.1002/jps.22552

Rodgers T, Rowland M (2006) Physiologically based pharmacokinetic modelling 2: Predicting the tissue distribution of acids, very weak bases, neutrals and zwitterions. J Pharm Sci 95(6):1238–1257, https://doi.org/10.1002/jps.20502

Rowland Yeo K, Walsky RL, Jamei M, Rostami-Hodjegan A, Tucker GT (2011) Prediction of time-dependent CYP3A4 drug-drug interactions by physiologically based pharmacokinetic modelling: Impact of inactivation parameters and enzyme turnover. Eur J Pharm Sci 43(3):160–173, https://doi.org/10.1016/j.ejps.2011.04.008

Ruan J, Yang M, Fu P, Ye Y, Lin G (2014) Metabolic activation of pyrrolizidine alkaloids: Insights into the structural and enzymatic basis. Chem Res Toxicol 27(6):1030–1039, https://doi.org/10.1021/tx500071q

Schweinoch D (2014) Prediction of the hepatic uptake clearance using a compartmental modelling approach based on in vitro assay data. PhD thesis, University of Potsdam

Shou M, Hayashi M, Pan Y, Xu Y, Morrissey K, Xu L, Skiles GL (2008) Modeling, prediction, and in vitro in vivo correlation of CYP3A4 induction. Drug Metab Dispos 36(11):2355–2370, http://doi.org/10.1124/dmd.108.020602

SPARC (2019) ARChem LLC. SPARC Automated Reasoning in Chemistry. http://www.archemcalc.com/sparc.html

Templeton IE, Houston JB, Galetin A (2011) Predictive utility of in vitro rifampin induction data generated in fresh and cryopreserved human hepatocytes, Fa2N-4, and HepaRG cells. Drug Metab Dispos 39(10):1921–1929, https://doi.org/10.1124/dmd.111.040824

Turner D, Rotami-Hodjegan A, Tucker G, Yeo K (2007) Prediction of nonspecific hepatic microsomal binding from readily available physicochemical properties. Drug Metabolism Reviews 38(S1)(162), https://www.certara.com/poster/prediction-of-non-prediction-of-non-specific-hepatic-microsomal-binding-from-readily-specific-hepatic-microsomal-binding-from-readily-available-physicochemical-properties/
